# xMSanalyzer: automated pipeline for improved feature detection and downstream analysis of large-scale, non-targeted metabolomics data

**DOI:** 10.1186/1471-2105-14-15

**Published:** 2013-01-16

**Authors:** Karan Uppal, Quinlyn A Soltow, Frederick H Strobel, W Stephen Pittard, Kim M Gernert, Tianwei Yu, Dean P Jones

**Affiliations:** 1 BimCore, School of Medicine, Emory University, Atlanta, GA, USA; 2 Department of Medicine, Division of Pulmonary, Allergy and Critical Care, Emory University, Atlanta, GA, USA; 3 Mass Spectrometry Center, Emory University, Atlanta, GA, USA; 4 Department of Biostatistics and Bioinformatics, Rollins School of Public Health, Emory University, Atlanta, GA, USA; 5 Clinical Biomarkers Laboratory, Emory University, Atlanta, GA, USA; 6 School of Biology, Georgia Institute of Technology, Atlanta, GA, USA

## Abstract

**Background:**

Detection of low abundance metabolites is important for de novo mapping of metabolic pathways related to diet, microbiome or environmental exposures. Multiple algorithms are available to extract *m/z* features from liquid chromatography-mass spectral data in a conservative manner, which tends to preclude detection of low abundance chemicals and chemicals found in small subsets of samples. The present study provides software to enhance such algorithms for feature detection, quality assessment, and annotation.

**Results:**

xMSanalyzer is a set of utilities for automated processing of metabolomics data. The utilites can be classified into four main modules to: 1) improve feature detection for replicate analyses by systematic re-extraction with multiple parameter settings and data merger to optimize the balance between sensitivity and reliability, 2) evaluate sample quality and feature consistency, 3) detect feature overlap between datasets, and 4) characterize high-resolution *m/z* matches to small molecule metabolites and biological pathways using multiple chemical databases. The package was tested with plasma samples and shown to more than double the number of features extracted while improving quantitative reliability of detection. MS/MS analysis of a random subset of peaks that were exclusively detected using xMSanalyzer confirmed that the optimization scheme improves detection of real metabolites.

**Conclusions:**

xMSanalyzer is a package of utilities for data extraction, quality control assessment, detection of overlapping and unique metabolites in multiple datasets, and batch annotation of metabolites. The program was designed to integrate with existing packages such as apLCMS and XCMS, but the framework can also be used to enhance data extraction for other LC/MS data software.

## Background

Liquid chromatography coupled with mass spectroscopy (LC/MS) is rapidly evolving as a method of choice for chemical phenotyping of biological systems. Targeted, non-targeted and hybrid methods provide effective means to detect and quantify a broad range of small molecules, including amino acids, lipids, sugars, drugs and environmental chemicals, in cells, tissues, or biofluids. Applications include metabolite profiling of tumor samples [1], identifying disease biomarkers [2], and studying complex biological networks [3].

High-resolution metabolomics uses high-resolution mass spectrometry to detect thousands of chemicals, both known and unidentified, as ions with high mass accuracy *m/z* and defined chromatographic retention time. The approach is flexible in that it allows fragmentation of selected ions for identification and stable isotope dilution for quantification of specific targets [4].

LC/MS methods are inherently limited by reproducibility of LC and MS, and numerous approaches have been used to improve both qualitative and quantitative reproducibility [5-8]. Lange et al. [8] reported that XCMS [9] performs better or comparable to other LC/MS alignment tools such as msInspect [10], MZmine [11,12], OpenMS [13,14], SpecArray [15], and XAlign [16] for the analysis of metabolomics data. XCMS [9] is a widely used R package for LC/MS data analysis, which incorporates novel nonlinear retention time alignment, matched filtration, peak detection, and peak matching. XCMS uses a second derivative Gaussian filter for feature detection and noise removal, and uses a feature-matching algorithm that performs binning of features by mass followed by use of a kernel density estimator to resolve groups of peaks with different retention time. A nonlinear retention time deviation profile is calculated for each sample using the dynamically identified endogenous metabolites as standards.

An adaptive processing method, apLCMS, was recently developed which contains a set of algorithms that improved processing of high-resolution LC/MS data [17]. Technical improvements included adaptive tolerance level searching rather than hard cutoff or binning, use of non-parametric methods to fine-tune intensity grouping, use of run filter to better preserve weak signals and use of model-based estimation of feature intensities for absolute quantification. The method involves 5 major processing steps: 1) noise filter, 2) feature identification, 3) retention time correction, 4) *m/z* feature alignment across multiple spectra, and 5) re-analysis to capture features originally missed because of weak signal relative to the signal to noise filter.

In the present study, we present xMSanalyzer, an R package with utilities for improving peak detection using existing methods such as apLCMS and XCMS, analyzing quality of metabolite data, finding overlapping and unique set of metabolites in two or more datasets, and annotating batches of metabolites in terms of matches to known compounds and pathways in databases such as Metlin, KEGG, HMDB, LipidMAPs, and PubChem. The results demonstrate that a data merger and quality filter scheme using systematic variation of parameter settings for peak detection allows detection of more features, thereby improving both the sensitivity and reliability of feature detection.

We examined the effect of varying parameter settings on data extraction using apLCMS as a basis for testing. The apLCMS routine has user-input parameters for the number of points to define a feature and the frequency of occurrence of a feature among consecutive scans. Because the scanning interval with Fourier-transform mass spectrometers is relatively constant, the optimal number of points to define a peak can change over the course of an LC run. xMSanalyzer varies the number of points and selects the most reproducible number to best define each peak. The results show that an increased number of metabolic features can be obtained by using an optimized feature detection routine, xMSanalyzer, while improving quantitative reproducibility. In addition, feature identification criteria for dietary and environmental chemicals, which may be present in only a small fraction of samples, are likely to differ from criteria used to extract information on higher abundance chemicals found in most samples [18]. xMSanalyzer can extract features present both in low abundance and in only a fraction of samples. The package has been developed to facilitate data analysis, comparison, and annotation of metabolomics data. To make it accessible with multiple softwares, we have developed xMSanalyzer to work with either apLCMS or XCMS.

## Implementation

### Program description

xMSanalyzer utilities can be classified into four main modules: 1) feature detection module to increase the number of quantitatively reproducible features by processing samples at two or more parameter settings, merging the resulting data, and selecting data based upon feature consistency, 2) sample quality module to support quality control analysis, 3) feature overlap module to detect overlap among multiple datasets or software packages and visualize the extent of overlaps, and 4) batch annotation module to facilitate annotation of metabolites.

### Software distribution and input requirements

xMSanalyzer is available for download at:

http://userwww.service.emory.edu/~kuppal2/xMSanalyzer/. The program depends on xcms (Bioconductor) or apLCMS (http://www.sph.emory.edu/apLCMS), XML (CRAN), R2HTML (CRAN), snow (CRAN) and limma (Bioconductor).

### Algorithm implementation

#### apLCMS.align: sample processing using apLCMS at one or more set of parameters

This utility in xMSanalyzer calls the *cdf.to.ftr()* function in the apLCMS package [17] and performs serial sample processing at multiple combinations of two parameters: min.run (minimum length of elution time for a series of signals grouped by *m/z* to be considered a feature; default value: 3) and min.pres (minimum proportion of scans in which the signal was present; default values: 0.3, 0.8). The function allows the user to define parameters such as min.exp (minimum number of samples in which a feature is present). This differs from the original apLCMS in that the original only allows one set of parameters, whereas this function allows multiple sets. The resulting tables containing *m/z*, retention time, and peak intensities in each sample are stored at each parameter combination.

#### XCMS.align: sample processing using XCMS at one or more set of parameters

The *XCMS.align* function in xMSanalyzer is a wrapper function based on the xcms Bioconductor package for preprocessing/analysis of mass spectral data. The *XCMS.align* utility performs serial sample processing at multiple combinations of four parameters: step (the step size; default values: 0.001, 0.01, 0.1), mzdiff (minimum difference for features with retention time overlap; default values: 0.001, 0.01, 0.1), snthresh (signal-to-noise ratio cutoff; default values: 3, 6, 10), and max (maximum number of peaks per EIC; default values: 5, 10). The resulting tables containing *m/z*, retention time, and peak intensities in each sample are stored at each parameter combination.

#### Evaluate.Samples: evaluating sample reproducibility

If at least two analytical replicates are present for each biological sample, this function calculates the mean pairwise Pearson correlation coefficient between sample replicates using the built-in *cor()* function in R. Only the features with no missing values are used to evaluate correlation. Analytical “replicates” refer to multiple injections from the same biological sample; whereas, “samples” refer to different biological samples.

#### Evaluate.Features: evaluating quantitative feature reproducibility across sample replicates

This utility uses the built-in *summary()* function in R to calculate the summary statistics of the Percent Intensity Difference (PID; two analytical replicates) or coefficient of variation (CV; more than two analytical replicates) as a statistical measure to evaluate feature consistency. PID is defined as percent ratio of absolute intensity difference to mean intensity, and coefficient of variation is defined as the ratio of the standard deviation to mean intensity. Only the samples with no missing values are used to evaluate PID if number of replicates is equal to two.

#### Merge.Results: merging features detected at multiple input parameter settings

We use a four-step process to merge features from different parameter settings. In step one, features detected at settings *P*_*1*_ and *P*_*2*_ are combined into one list. In step two, features are grouped by a user-defined *m/z* tolerance (5 ppm is appropriate for high resolution MS but may not be suitable for lower resolution instruments; for the LTQ-FT/MS, examination of *m/z* tolerance shows little difference between 5 and 10 ppm) (Additional File 1). In step three, features are further sub-grouped based on a user-defined retention time tolerance. Users are recommended to use the *find.Overlapping.mzs* function below to optimize the retention time tolerance threshold. In step four, a paired t-test is used to compare the intensity levels of the metabolites only for the redundant features that have *m/z* and retention time within defined tolerance levels as described above. Users should note that there is no default correction for multiple comparisons as the *t*-statistic is user defined. Features with minimum median PID (or median CV; for more than two technical replicates) are chosen as representatives of each sub-group, and added to the final list. This scheme allows identification of unique features, and selection of the most consistent feature as a representative for features that overlap.

#### Find.Overlapping.mzs: m/z-based feature matching across multiple datasets

The *find.Overlapping.mzs* function operates on two sets of feature lists with *m/z* and retention times for each feature, denoted by *L*_*1*_ and *L*_*2*_, and iterates over all *m/z* values in *L*_*1*_ to find those that are within a user defined *m/z* (ppm) and retention time (sec) threshold in *L*_*2*_. Optionally, the user can match features based on only the *m/z* values by setting time.thresh = NA. The *find.Unique.mzs* function uses a similar algorithm to find unique features that are not within a user defined mass and retention time threshold level.

#### GetVenn: visualize feature matching results

This utility calls the *find.Overlapping.mzs* and *find.Unique.mzs* functions and generates a Venn diagram showing the extent of overlap between datasets (up to three).

#### check.mz.in.replicates: Metabolic characteristics of individuals

This utility allows identification of rare features that are present in only some biological samples, but are present in majority of the analytical replicates of individual samples as a result of unique environmental exposure. The min.samps and min.reps are user defined values for defining the minimum number of samples and minimum percentage of replicates in which a feature should be detected.

#### Feat.Batch.Annotation: characterization of metabolites

This utility uses the *readHTMLTable()* function in the XML package in R to parse the list of compounds and pathways IDs from METLIN and KEGG REST interface available at: http://metlin.scripps.edu/metabo_list.php and http://www.kegg.jp/kegg/rest/keggapi.html, respectively. The output is generated as an HTML report and a text file that includes pathway and compound annotations with links to external databases such as Metlin, KEGG Compound, KEGG Pathway, PubChem Compound, PubChem Substance, HMDB, ChEBI, CAS, and LipidMAPS. The function takes as input a data frame with a list of input *m/z*, a user-defined *m/z* threshold (ppm) to define the minimum and maximum mass range, list of adducts; eg: c(“M + H”, “M + H-H2O”), and the output folder location. A sample annotation report is available at the software homepage: http://http//userwww.service.emory.edu/~kuppal2/xMSanalyzer/SampleAnnotation.html.

### xMSwrapper

**Figure 1:**
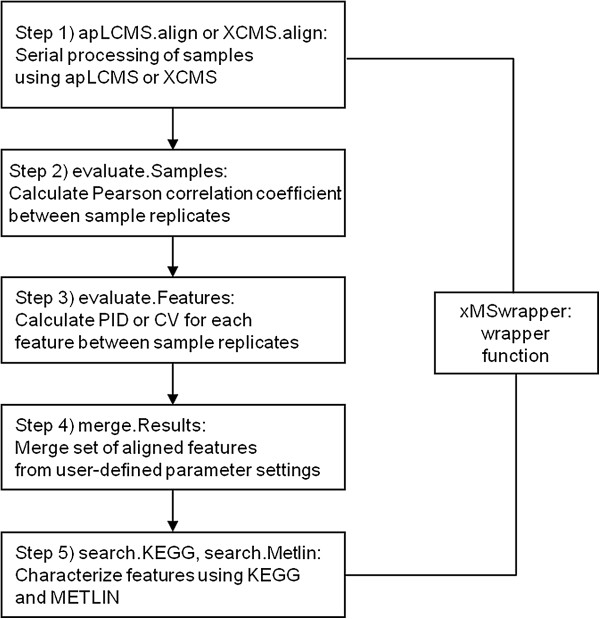
xMSwrapper workflow.

The wrapper function includes five steps as shown in Figure 1 in which data are extracted with different parameters to maximize feature detection, evaluated for sample quality, evaluated for feature consistency, merged to obtain a combined feature table, and characterized with known metabolites and pathways. Users have the option to filter poor quality samples and features based on correlation between technical replicates and feature reproducibility measures such as PID or CV, respectively.

## Results and discussion

To illustrate the applications of xMSanalyzer, metabolomics data were derived from two samples sets of human plasma. Sample Set 1 consisted of 22 samples taken from a dietary restriction study [19]. Sample Set 2 was human reference samples from two sources; one reference sample was donated from the National Institute of Standards and Technology (NIST). The second reference sample consisted of pooled plasma from Equitech-Bio, Inc (Kerrville, TX). Detailed procedure for sample extraction and metabolomics analysis is described in a recent technical manuscript [20]. For Sample Set 1, analyses were performed alternately between two anion exchange (AE) columns (column A and B) to compare characteristics on two columns [21]. For Sample Set 2, analyses were performed alternately using AE and reverse phase (C_18_) columns [20]. Mass spectral data were collected with a Thermo LTQ-FT mass spectrometer (Thermo Fisher, San Diego, CA) set to collect data from *m/z* 85 to 850 as described [21] with mass resolution of 50,000. Data were stored as .raw files and converted using Xcalibur file converter software (Thermo Fisher, San Diego, CA) to .cdf files for further data processing.

The software was developed in three stages. First, the methodology was designed and tested using apLCMS. Second, the software was written to be used in conjunction with apLCMS. Lastly, the software was adapted to be integrated with XCMS.

### Quantitative evaluation

**Figure 2:**
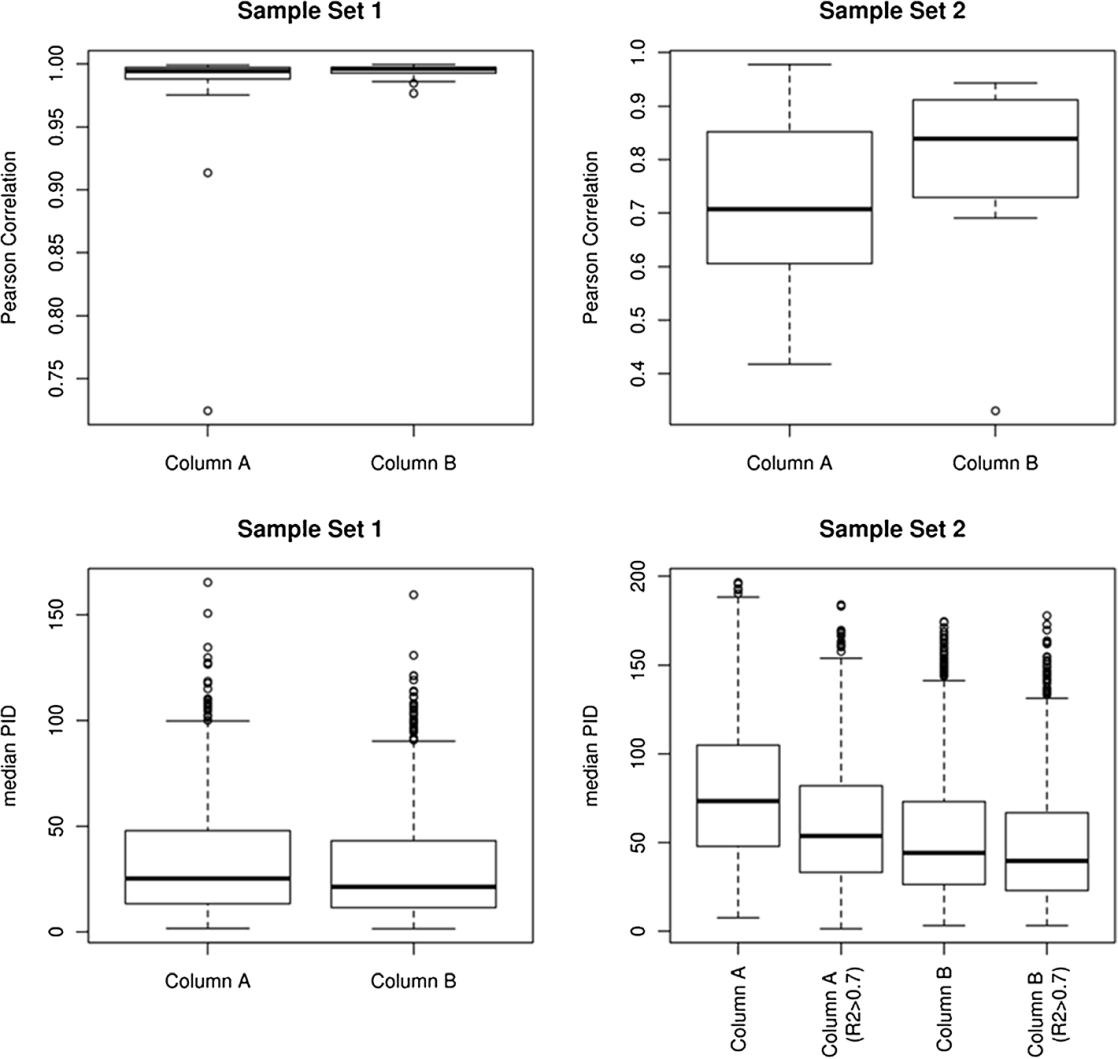
**Quantitative evaluation of LC/MS profile alignment using apLCMS. **Top row shows Pearson correlation within sample duplicates in both datasets; bottom row shows the median PID of feature intensities within sample duplicates. The effect of re-aligning profiles after removing poor quality samples (correlation coefficient, R^2^ < 0.7) on the quantitative reproducibility of features is shown in the bottom right panel. A noticeable difference in median PID can be seen between alignment using all samples and alignment using only high quality samples for both columns of Sample Set 2.

There are various sources of variability such as biological, sample processing and instrumental that can affect the quality of alignment of LC/MS profiles. Identification of technical variation within sample replicates is critical to minimize false positives as this could influence the downstream analysis of biological variations between different samples, which is of main interest. Pairwise Pearson correlations of feature intensities can be used as a metric for assessing process variability and estimate the overall difference in feature intensities between aligned replicates [6]. xMSanalyzer uses the functions *evaluate.Samples* and *evaluate.Features* to calculate the mean pairwise Pearson correlation coefficient between sample replicates, as shown in Figure 2. The higher correlation of feature intensities between sample replicates of Sample Set 1 indicates good sample quality and less technical variation within same experiment. However, low correlation between replicates in Sample Set 2 indicates a potential technical variability or poor sample quality. The effect of realigning profiles after removing poor quality samples (correlation coefficient, R^2^ < 0.7) on the quantitative reproducibility of features is shown in the bottom right panel of Figure 2. A noticeable difference in median PID can be seen between alignment using all samples and alignment using only high quality samples for both columns of Sample Set 2. The *samp.filt.thresh* and *feat.filt.thresh* parameters in xMSanalyzer allow the users to define thresholds to retain only high quality samples and features for downstream analysis.

### Parameter sensitivity analysis

Sensitivity analysis was performed to identify the parameters that affect the number of features detected and the quantitative reproducibility when requiring a feature to be present in at least 50% of the LC/MS profiles. Sensitivity analysis is the assessment of the relationship between the input parameters and the output [7]. The qualitative or quantitative effect of systematic variation of parameter *X*_*i*_ on output *Y* is studied while all other parameters are fixed. For apLCMS, we focus on the evaluation of four parameters that are used in the feature detection process for grouping of data points based on *m/z* (max.bw and min.bw) or noise removal (min.run and min.pres): min.run (minimum length of elution time for a series of signals grouped by *m/z* to be considered a feature), min.pres (minimum proportion of scans in which the signal was present), min.bw (minimum bandwidth of kernel smoother fitted along time axis to determine whether there is one single feature or multiple features), and max.bw (maximum bandwidth of kernel smoother fitted along time axis to determine whether there is one single feature or multiple features). The number of identified features and the median PID (from sample duplicates) were used as sensitivity measures and are compared below.

### Feature detection optimization

Some metabolites have narrow elution peaks, while others have broader peaks. The accuracy of peak integration is dependent on the parameters used to define the peaks. The tradeoff between feature detection sensitivity and reliability could be balanced by merging unique features detected at individual parameter settings and selecting more reproducible features from the overlapping ones, respectively. The results in Table 1 suggest that the merge algorithm implemented in xMSanalyzer increases sensitivity of feature detection (Features; 2^nd^ column) without compromising for the reliability (mPID; 3^rd^ column) of the features. To do this, xMSanalyzer uses the function *merge.Results,* which is a four-step process to merge features detected at settings *P*_*1*_ and *P*_*2*_, where *P*_*1*_ = *P*[min.run (*x*_*1*_), min.pres (*y*_*1*_)] and *P*_*2*_ = P[min.run (*x*_*2*_), min.pres (*y*_*2*_)] , as described in the Implementation section. The selection of optimal *P*_*1*_ and *P*_*2*_ are described in the following sections.

**Table 1:** Evaluating fitness of parameter combinations based on parameter sensitivity analysis

**Parameter**^**a**^	**Features**	**mPID**^**b**^	**S**^**c**^ = N-(30*mPID)	**S**^**d**^ = N-(100*mPID)
12, 0.5	1454	33.12	460.4	−1858
3,0.3	2350	39.75	1157.5	−1625
3,0.5	1940	34.72	898.4	−1532
3,0.8	1653	30.60	735	−1407
3,0.3 υ 3,0.5	2363	36.16	1278.2	−1253
3,0.3 υ 3,0.8	2384	35.69	1313.3	−1185
3,0.5 υ 3,0.8	2022	32.22	1055.4	−1200
3,0.3 υ 12,0.5	2310	36.59	1214.4	−1342
3,0.5 υ 12,0.5	2037	33.40	1035	−1303
3,0.8 υ 12,0.5	1816	30.37	904.9	−1221

Data was taken from Column A of Sample Set 1. ^a^Only a subset of the results is shown in the table. ^b^Median PID (mPID) averaged over all features. ^c^Scoring function weighs more importance to number of features; ^d^Scoring function weighs more importance to quality of features.

### Increased leniency in feature detection within a sample by decreasing min.run

**Figure 3:**
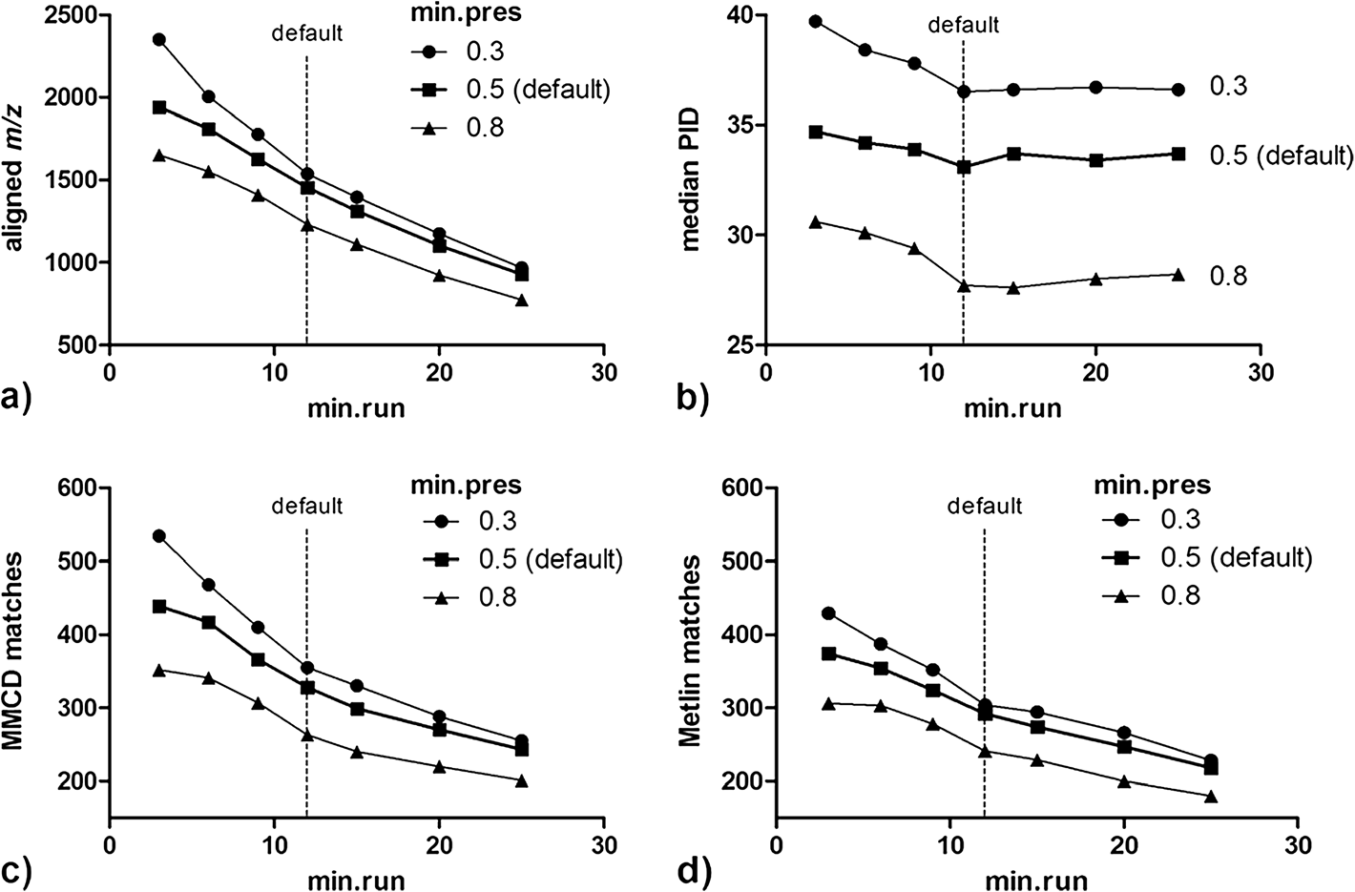
**Variation in stringency for feature detection in sample analyses. **Using apLCMS, min.run was varied from 25, 20, 15, 12, 9, 6, 3 (panel **a**); min.pres was varied from 0.3, 0.5, 0.8 (panel **b**); and *m/z* were matched to Madison Metabolomics Consortium Database (MMCD) (panel **c**) and Metlin database (panel **d**) for Column A from Sample Set 1 at 5 and 10 ppm mass tolerance. Results at 10 ppm tolerance level are shown here.

Using the default settings as a control condition (min.run = 12; min.pres = 0.5), we used Sample Set 1 to test whether more lenient min.run, varied from 25 to 20, 15, 12, 9, 6, and 3, increased the number of features detected by apLCMS at min.exp = 50%, i.e., a feature is included if present in at least 50% of the profiles. The results (Figure 3a) showed that the default setting (feature detected in 50% of 24 scans) detected 1454 features with a median PID of 33.12%. Decreasing stringency in min.run resulted in a progressive increase in the number of features (Figure 3a and Additional File 2). Comparison of the features detected with different parameters using the xMSanalyzer function *find.Overlapping.mzs* showed that each analysis identified a set of features that overlapped with those of the default settings (Additional File 3) with small change in median PID from duplicates (Figure 3b). The finding that the PID between analyses did not increase by a large extent as stringency in min.run was decreased indicates that in this high-throughput operation, narrower peaks were detected with reproducibility similar to broader peaks. This is important because it suggests that the criterion for 25 points to define a chromatographic peak may be excessively stringent for high throughput analyses.

### Effect of variation in min.pres on detection and PID of features

To determine whether decreased stringency in min.pres would similarly improve detection, we compared results obtained with min.pres varied from 0.8 to 0.5 and 0.3 (Figure 3a and Additional File 2). As compared to the default setting, additional features were detected at less stringent setting (min.pres = 0.3; feature detected in 30% of 24 scans, Figure 3a), while the consistency of the features decreased (Figure 3b). Increasing the threshold of minimum proportion of signal presence in a segment to consider it as a feature (min.pres = 0.8; feature detected in 80% of 24 scans, Figure 3a) led to decline in the number of features detected, but there was a notable improvement in PID indicating that higher min.pres improved quantitative reliability of detection (Figure 3b). A similar pattern was observed at all min.run variations.

### Effect of variation in kernel smoother bandwidth on feature detection

The min.bw parameter was varied from 1 to 5, 30, and NA (estimated from data), and max.bw was varied from 30 to 60, and NA (estimated from data) at default setting (feature detected in 50% of 24 scans), but as shown in Additional File 4, no effect on the number of features detected was observed.

The results show that the min.pres and min.run parameters used in the feature detection step of apLCMS have a significant effect on sensitivity of feature detection and quantitative reproducibility of the features. The results in Figure 3 indicate that increase in number of features detected by relaxing these parameters reduces the consistency of the features to some extent. The features detected at each parameter setting were searched against databases of known metabolites such as Madison Metabolomics Consortium Database (MMCD; http://mmcd.nmrfam.wisc.edu/) [22] and Metlin Metabolite Database (http://metlin.scripps.edu/) [23] at 5 and 10-ppm mass tolerance levels. As compared to the default setting, additional matches to known metabolites were obtained using the less stringent settings suggesting that the low abundance peaks that were detected are possibly real chemicals (Figure 3c, d; results at 10 ppm). Comparable pattern was observed at 5 ppm mass tolerance (not shown).

### Identification of optimal pair of parameter settings using parameter optimization

An optimal pair of parameter settings was determined such that merging the results from the two settings, *P*_*1*_ υ *P*_*2,*_ using the *merge.Results* function of xMSanalyzer resulted in identification of maximum number of features with minimum overall PID between duplicates. To do this, a scoring function (1) was designed to evaluate the performance of each setting based on the number of features detected and the quantitative reproducibility of the features (i.e., PID). The optimization algorithm searches over a defined set of individual and paired parameter settings, and selects the combination, *P*_*1*_ υ *P*_*2*_, that maximizes the scoring function.

(1) argmaxN-w*medianPID

$$P\left(x_{1},y_{1}\right)\mathrm{\upsilon P}\left(x_{2},y_{2}\right)$$

such that,

$$\begin{array}{l} x_{1},x_{2}\;\varepsilon \;\left\{3,6,9,12,15,20,25\right\},\\ y_{1},y_{2}\;\varepsilon \;\left\{0.3,0.5,0.8\right\},\\ w\;\varepsilon \;\left\{30,100\right\} \end{array}$$

where

*x*_*1*_*,x*_*2*_: search space for min.run parameter,

*y*_*1*_*,y*_*2*_: search space for min.pres parameter,

*P*(*x*_*1*_*,y*_*1*_) : feature alignment results at min.run = x_1_, and min.pres = y_1_,

*P*(*x*_*2*_*,y*_*2*_) : feature alignment results at min.run = x_2_, and min.pres = y_2_,

*N*: number of aligned features after merge,

w: weighting parameter (arbitrary constant),

median PID: median % Intensity Difference averaged over all features

The weighting parameter, *w*, in the scoring function balances the importance between the quantity and quality of features such that a higher score is assigned to settings that satisfy the criteria of higher number of features and lower variability in the intensity levels between sample duplicates. Running the apLCMS algorithm multiple times linearly increased the computation time while the number of features detected reached a saturation point following the union of the two parameter settings, *P*_*1*_ and *P*_*2*_ (Additional File 5).

Each parameter set (e.g. 3,0.5) and the union of two parameter sets (e.g. 3,0.3 υ 3,0.5) were evaluated using the scoring function described above that accounts for the number of features (Table 1, column 4) and the quality of features (Table 1, column 5) for sensitivity analysis. The analysis was performed on both datasets, and {3,0.3 υ 3,0.8} provided the largest number of features without compromising quantitative reproducibility in all cases (Table 1 and Additional File 6). Users can use a similar approach to determine optimal parameter settings for their datasets either by using the scoring method presented here, or by taking into account additional criteria such as number of internal standards detected.

### xMSanalyzer

**Figure 4:**
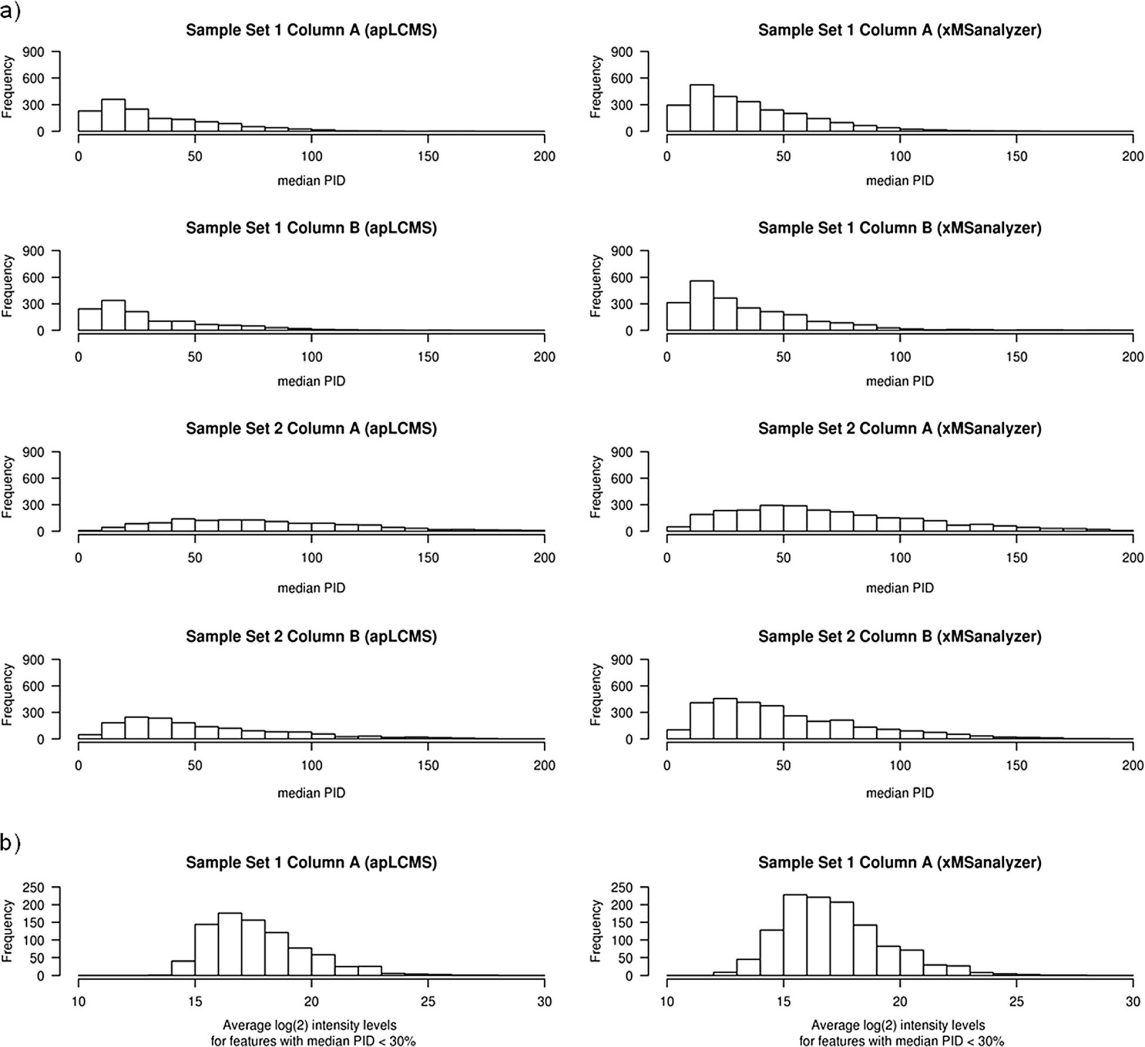
**xMSanalyzer improves the sensitivity of feature detection without compromising data quality. a**) Histograms showing number of peaks with ranges of percent intensity differences (PID) for LC/MS profile alignments using apLCMS (left) and xMSanalyzer (right). The results show that the xMSanalyzer routine allows detection of more quantitatively reproducible features; **b**) Histograms showing the average log2 intensity levels in features with median PID less than 30% detected using apLCMS (left) and xMSanalyzer (right) in Sample Set 2, Column A. xMSanalyzer not only improves the overall quantitative reproducibility of features, but also allows detection of reliable low abundance features.

Based upon these analyses, an optimized data extraction routine, xMSanalyzer, was developed with code provided in R in Additional File 7. The performance of xMSanalyzer was evaluated on all datasets with respect to apLCMS, and significant improvement in the number of quantitative reproducible features was observed as shown in Figure 4a. Additionally, xMSanalyzer improved the sensitivity of feature detection by capturing more low abundance features (Figure 4b).

The xMSanalyzer routine is designed to allow the users to define which parameter settings should be merged according to desired priorities for feature extraction versus reliability, or both. The default setting of {3,0.3 υ 3,0.8} gives higher precedence to the number of features; however, the users can select other options such as {3,0.5 υ 3,0.8} or {12,0.5 υ 3,0.8} that give importance to quality as well. The effect of input parameters on the performance of xMSanalyzer is illustrated in Table 2. On average, xMSanalyzer identified 410 (73.2%) and 319 (57%) more quantitatively reproducible features (median PID <30%) at {3,0.3 υ 3,0.8} and {12,0.5 υ 3,0.8}, respectively. However, the overall data quality was better at {12,0.5 υ 3,0.8}.

**Table 2:** Comparison of the number of quantitative reproducible features between apLCMS and xMSanalyzer

**Datasets**	**apLCMS**	**xMSanalyzer**	**xMSanalyzer**
	**P{12,0.5}: default**	**P**_**1**_**{3,0.3} υ P**_**2**_**{3,0.8}**	**P**_**1**_**{12,0.5} υ P**_**2**_**{3,0.8}**
Sample Set 1 (Column A)	839 out of1454 (57.7%)	1208 out of 2384 (50.6%)	1115 out of 1816 (61.3%)
Sample Set 1 (Column B)	791 out of1238 (63.89%)	1236 out of 2201 (56.1%)	1081 out of 1615 (66.9%)
Sample Set 2 (Column A)	134 out of1324 (10.1%)	470 out of 2677 (17.5%)	424 out of 2256 (18.7%)
Sample Set 2 (Column B)	474 out of1573 (30.1%)	966 out of 2969 (32.5%)	897 out of 2546 (35.2%)
Average over all datasets	560 (40%)	970 (37.9%)	879 (42.7%)

The number of reproducible features (median PID < 30%) identified by apLCMS at min.run = 12 and min.pres = 0.5 and xMSanalyzer at P_1_{3,0.3} υ P_2_{3,0.8} that weighs more importance to the number of features as compared to quality, and at P_1_{12,0.5} υ P_2_{3,0.8} that gives balanced importance to the quality and quantity of features.

### Comparison of xMSanalyzer with apLCMS and XCMS

The performance of using xMSanalyzer in conjunction with apLCMS was compared to XCMS [9] and apLCMS [17]. The feature alignment of both sample sets used in our study was performed at all combinations of the parameters in XCMS, and the results of the best parameter settings were used for comparison. Settings for all three algorithms, apLCMS, xMSanalyzer, and XCMS, required the features to be detected in at least 50% of the profiles. The apLCMS routine was compared using default settings (min.pres = 0.5, min.run = 12) and provided more features than XCMS by an average of 19.3% (Table 3). The apLCMS routine is designed for high-resolution mass spectrometers, so accurate *m/z* may be more clearly separated from noise than in XCMS. This would provide superior capture of weak peaks. xMSanalyzer, which combined variations in the parameter settings, increased the average number of features detected in two different sample sets compared to apLCMS and XCMS, by 83.1% and 118%, respectively. An example of three features recognized by xMSanalyzer, but missed by both apLCMS and XCMS, is shown in Additional File 8.

**Table 3:** Comparison of the number of features detected (total and known) using apLCMS, xMSanalyzer, and XCMS

**Datasets**	**apLCMS**	**xMSanalyzer-apLCMS**	**XCMS v1.20.1**
	**{default}**	**{3,0.3} υ {3,0.8}**	**{step = 0.001, snthresh = 3, max = 5, mzdiff = 0.1}**
Sample Set 1 (Column A)	1454	2384	1027
	MMCD: 314 (21.6%)	MMCD: 534 (22.3%)	MMCD: 222 (21.6%)
	Metlin: 292 (20.1%)	Metlin: 433 (18.1%)	Metlin: 230 (22.4%)
Sample Set 1 (Column B)	1238	2201	998
	MMCD: 309 (25%)	MMCD: 557 (25.3%)	MMCD: 261 (26.1%)
	Metlin: 279 (22.5%)	Metlin: 468 (21.2%)	Metlin: 252 (25.2%)
Sample Set 2 (Column A)	1324	2677	1262
	MMCD: 408 (30.8%)	MMCD: 732 (27.3%)	MMCD: 324 (25.7%)
	Metlin: 497 (37.5%)	Metlin: 705 (26.3%)	Metlin: 431 (34.2%)
Sample Set 2 (Column B)	1573	2969	1395
	MMCD: 508 (32.3%)	MMCD: 794 (26.7%)	MMCD: 359 (25.7%)
	Metlin: 693 (44.1%)	Metlin: 848 (28.5%)	Metlin: 514 (36.8%)
Average over all datasets	Total: 1397	Total: 2558	Total: 1171
	Known metabolites: 413 (29.6%)	Known metabolites: 634 (24.8%)	Known metabolites: 324 (27.7%)

To give a reference to the number of *m/z* that produce potential matches to known metabolites, all *m/z* identified by each routine were searched against the [M + H] + adducts of the known metabolites using MMCD and Metlin at 5 and 10-ppm mass tolerance level. As previously stated, comparable results were obtained at the two mass tolerance levels. The results show that both databases retrieved comparable numbers of matches for *m/z* detected by each routine (Table 3). The xMSanalyzer algorithm obtained a higher number of matches than both apLCMS and XCMS for both sample sets; 634 using xMSanalyzer compared to 413 using apLCMS and 324 using XCMS. However, the apLCMS routine found the highest average percentage of matches (29.6%) compared to xMSanalyzer (24.78%) and XCMS (27.7%). When xMSanalyzer was run in conjunction with XCMS, the average number of features detected over all datasets increased from 1171 (XCMS alone; Table 3) to 1771 (XCMS with xMSanalyzer; data not shown).

Using similar search criteria, we annotated a list of 20 randomly generated features within 85–850 *m/z* range using Metlin. The process was repeated six times, and on an average, 12.5% of the features found hits in the Metlin database. This suggests that true identity of metabolites cannot be established by database searches as previously reported [24]; however, the results in Figure 4 and Table 3 suggest that xMSanalyzer allows detection of an increased number of metabolites (known and unknown) that can then be targeted for experimental validation.

### Experimental validation

To test if xMSanalyzer was improving feature extraction by generation of false positives, a list of features was randomly selected from those features that were detected exclusively by xMSanalyzer and not by XCMS or apLCMS in Sample Set 2. From these, we arbitrarily selected 17 features that had MS/MS spectra available in the Metlin database (Additional File 9) and used these for MS/MS on an LTQ-Velos Orbitrap (Thermo Scientific, San Jose, CA, USA) at isolation width of 1 a.m.u for ion trap and 2 a.m.u for Orbitrap. Normalized collision energy of 40% in collisional-induced dissociation (CID) with 10 ms activation time was used to acquire MS/MS spectra. A cycle of one full scan followed by 2 CID MS/MS scans was acquired for targeted ions and repeated continuously through each elution time ±60 s for each feature. All 17 features were identified by chromatographic peaks within 60 s of the predicted elution time (Additional Files 9, 10 and 11). Of the 17 features, 14 eluted as single peaks, two (*m/z* 340.1607 and *m/z* 389.2494) appeared as multiple peaks, and one (*m/z* 337.235) eluted across a wide range of time that was consistent with one of the solvents used for the chromatography (Additional File 11). Similar fragmentation patterns were obtained from MS/MS experiments from the ion trap and Orbitrap indicating that the peaks were not electronic background noises.

Comparison of MS/MS spectra to those in the Metlin database was consistent with identity of three features as putative metabolites in the Metlin database (Additional File 10). For further confirmation, we used DeconMSn v2.2.2.2 [25], which deisotopes the precursor ion isotopic profiles to determine monoisotopic masses of parent ions, and produces. DTA text files of the fragment ion spectra. 11 out of 17 features (including the features with matches in Metlin MS/MS database) were identified as putative precursor ions (Additional Files 10 and 11). Although one caveat of using this approach is that some parent ions could have overlapping elution profiles with other ions present in the isolation width resulting in deconvolution ambiguity. Interestingly, 10 out of the 11 putative metabolites had first quartile PID less than 30% (Additional File 9) suggesting that the PID or CV measures can be used for eliminating false positives.

To further test the utility of xMSanalyzer for detection of low-level chemicals, we analyzed samples from a population of dialysis patients who are more likely to be exposed to environmental agents through pharmaceuticals, water, and plastics than a healthy population. Analysis of 10 biological samples, each with two analytical replicates, using apLCMS at min.exp = 2 samples resulted in detected of over 15,900 features and 9,300 features on the AE and C18 columns, respectively (Table 4). This result more than doubled the number of features detected by apLCMS at the default settings, and two-thirds of the *m/z* detected did not match to metabolites in the MMCD database.

**Table 4:** xMSanalyzer doubles the number of features detected in human patient population

**Parameter**	**AE column**	**C18 column**
12,0.5 (default)	6538	4337
	MMCD: 2412	MMCD: 1556
3,0.3	14004	10729
	MMCD: 4206	MMCD:2795
3,0.8	10837	8069
	MMCD: 3624	MMCD:2367
3,0.3 υ 3,0.8	15955	9396
(xMSanalyzer)	MMCD: 5579	MMCD: 2819

Dialysis patients are highly susceptible to environmental chemicals due to repeated exposures to pharmaceuticals, water, and plastics during dialysis sessions. A dataset from 10 plasma samples collected in dialysis patients, each with 2 analytical replicates, were analyzed by xMSanalyzer (at min.exp = 2) and resulted in a >2-fold increase in feature detection (from 6538 to 15955 features on the AE column), in which most *m/z* are unmatched in the metabolomics database MMCD.

Accurate identification of metabolites remains a challenge in metabolomics; however, incorporation of reliable low abundance and variable peaks (which would have been missed otherwise) should assist in the discovery of new metabolites and identification of parent compounds. xMSanalyzer broadens the range of feature detection and therefore may facilitate detection and future identification of currently unidentified but important chemicals, such as low-level environmental chemicals and products of enteric flora.

## Conclusions

Most LC/MS data extraction programs are designed to identify peaks in a conservative manner, which tends to preclude detection of low abundance chemicals and chemicals found in small subsets of samples. xMSanalyzer comprises a package of utilities for metabolomics data that can be integrated with existing packages such as apLCMS and XCMS to improve detection of low abundance and variable peaks from high resolution metabolomics data, assess feature and sample quality within technical replicates, compare two or more datasets, and perform batch annotation of metabolites with known chemicals and biological pathways. The optimization algorithm compares favorably to the stand-alone versions of apLCMS and XCMS by increasing the number of quantitatively reproducible features. Application of optimization routines like xMSanalyzer to high resolution metabolomics data will likely enhance metabolomics databases by allowing inclusion of *m/z* currently unidentified, such as dietary and environmental chemicals.

## Availability and requirements

**Project name:** xMSanalyzer

**Project home page:**http://userwww.service.emory.edu/~kuppal2/xMSanalyzer/

**Operating system(s):** Platform independent

**Programming language:** R

**Other requirements:** apLCMS or xcms, XML, R2HTML, limma, snow (R packages)

**License:** GNU GPLv2

**Any restrictions to use by non-academics:** none

## Competing interests

The authors declare that they have no competing interests.

## Authors’ contributions

KU, QAS and DPJ conceived and coordinated the software design; KU developed the software with advice from QAS, KMG, WSP, and DPJ; QAS generated metabolomics data for algorithm development; FHS reviewed the MS/MS data; KU analyzed the data with advice from QAS, KMG, FHS, and DPJ; WSP set up the computational environment for data transfer and analysis; KU drafted the manuscript with significant contributions from TY, QAS, FHS, and DPJ and the content was approved by all authors. All authors read and approved the final manuscript.

## Supplementary Material

Additional file 1 xMSanalyzer results at different +/− m/z tolerance levels (ppm) for merging features identified at {3,0.3} and {3,0.8} (Sample Set 1, 44 samples, min.exp = 50%). Click here for file

Additional file 2 **Feature detection using apLCMS while varying min.run and min.pres in a) Sample Set 1 Column B; b) Sample Set 2 AE Column; and c) Sample Set 2 C**_18_**Column.** Click here for file

Additional file 3 **Venn Diagrams representing overlapping features between the default setting and variations in min.run at min.pres = 0.3. *Only unique features at*m/z*tolerance level of 10 ppm were used to generate Venn diagrams using BioVenn (***http://www.cmbi.ru.nl/cdd/biovenn/***).*** Click here for file

Additional file 4 Effect of variation in min.bw and max.bw on feature detection at default settings using a random subset of 10 samples from the Sample Set 1 Column A. Click here for file

Additional file 5 Results for merging more than two parameter settings using the Sample Set 1 Column A. Click here for file

Additional file 6 Evaluating fitness of parameter combinations based on parameter sensitivity analysis in a) Sample Set 1 Column B; b) Sample Set 2 Column A; and c) Sample Set 2 Column B. Click here for file

Additional file 7 R code for xMSanalyzer. Click here for file

Additional file 8 **Extracted ion chromatograms of unique features identified by xMSanalyzer in three biological samples: a) *m/z *290.1358 (Gly-Asp-Val); b) *m/z *425.16421 (Asp-Tyr-Gln); c) *m/z *175.1187 (arginine).** Click here for file

Additional file 9 Summary of MS/MS analysis. Click here for file

Additional file 10 **MS/MS validation results for the metabolites exclusively identified by xMSanalyzer and with matches in Metlin. **The first column is the full MS scan, second column is the MS/MS spectrum on LTQ Velos Orbitrap, and the third column shows the corresponding MS/MS spectrum from Metlin’s database. Click here for file

Additional file 11 **MS/MS validation results for the 8 putative metabolites identified using DeconMSn. **The first row shows the elution profile of the feature, middle row shows the full MS scan, and the last row shows the deconvoluted MS/MS spectrum obtained from DeconMSn. Click here for file
