# Supplementary material for: xMSanalyzer: automated pipeline for improved feature detection and downstream analysis of large-scale, non-targeted metabolomics data
Source: BMC Bioinformatics. 2013 Jan 16;14:15. doi: 10.1186/1471-2105-14-15 (PMC3562220; doi:10.1186/1471-2105-14-15)
Supplement: Additional file 1 — xMSanalyzer results at different +/− m/z tolerance levels (ppm) for merging features identified at {3,0.3} and {3,0.8} (Sample Set 1, 44 samples, min.exp = 50%). [file 1471-2105-14-15-S1.doc]

**Additional File 1.** xMSanalyzer results at different +/- *m/z* tolerance levels (ppm) for merging features identified at {3,0.3} and {3,0.8} (Sample Set 1, 44 samples, min.exp=50%)

| **+/- mz tolerance level (ppm) for merging {3,0.3} and {3,0.8}** | **Number of features** | **Average median PID** | **Number of features with median PID <30%** |
| --- | --- | --- | --- |
| 1 | 2559 | 36.58 | 1266 |
| 5 | 2412 | 35.91 | 1213 |
| 10 | 2384 | 35.69 | 1208 |
| 20 | 2367 | 35.53 | 1203 |
