# Supplementary material for: xMSanalyzer: automated pipeline for improved feature detection and downstream analysis of large-scale, non-targeted metabolomics data
Source: BMC Bioinformatics. 2013 Jan 16;14:15. doi: 10.1186/1471-2105-14-15 (PMC3562220; doi:10.1186/1471-2105-14-15)
Supplement: Additional file 2 — Feature detection using apLCMS while varying min.run and min.pres in a) Sample Set 1 Column B; b) Sample Set 2 AE Column; and c) Sample Set 2 C18Column. [file 1471-2105-14-15-S2.doc]

**Additional File 2a.** Feature detection using apLCMS for Sample Set 1 study Column B samples.

| **Parameter Setting** | **Number of aligned *m/z* features** | **median PID between duplicates** |
| --- | --- | --- |
| a) Variation in min.run at min.pres = 0.3 | | |
| 25 | 834 | 34.27 |
| 20 | 998 | 34.77 |
| 15 | 1213 | 34.23 |
| 12 (default) | 1347 | 34.21 |
| 9 | 1541 | 34.52 |
| 6 | 1779 | 35.48 |
| 3 | 2112 | 36.85 |
| b) Variation in min.run at min.pres = 0.5 (default) | | |
| 25 | 801 | 31.40 |
| 20 | 951 | 31.99 |
| 15 | 1092 | 30.85 |
| 12 (default) | 1238 | 30.31 |
| 9 | 1418 | 30.67 |
| 6 | 1660 | 32.06 |
| 3 | 1800 | 32.55 |
| c) Variation in min.run at min.pres = 0.8 | | |
| 25 | 640 | 24.17 |
| 20 | 754 | 23.43 |
| 15 | 952 | 23.11 |
| 12 (default) | 1068 | 23.08 |
| 9 | 1258 | 24.38 |
| 6 | 1386 | 25.72 |
| 3 | 1470 | 27.02 |
| d) xMSanalyzer | | |
| 3, 0.3 ∪ 3,0.8 | 2201 | 33.85 |

**Additional File 2b.** Feature detection using apLCMS for Sample Set 2 samples from Column A.

| **Parameter Setting** | **Number of aligned *m/z* features** | **median PID between duplicates** |
| --- | --- | --- |
| a) Variation in min.run at min.pres = 0.3 | | |
| 25 | 775 | 69.59 |
| 20 | 942 | 70.23 |
| 15 | 1229 | 71.14 |
| 12 (default) | 1416 | 71.81 |
| 9 | 1652 | 74.05 |
| 6 | 2052 | 75.28 |
| 3 | 2618 | 76.14 |
| b) Variation in min.run at min.pres = 0.5 (default) | | |
| 25 | 726 | 67.74 |
| 20 | 861 | 69.43 |
| 15 | 1165 | 69.06 |
| 12 (default) | 1324 | 69.49 |
| 9 | 1574 | 71.74 |
| 6 | 1923 | 73.53 |
| 3 | 2510 | 74.29 |
| c) Variation in min.run at min.pres = 0.8 | | |
| 25 | 571 | 63.77 |
| 20 | 714 | 65.08 |
| 15 | 981 | 66.30 |
| 12 (default) | 1108 | 66.60 |
| 9 | 1330 | 69.34 |
| 6 | 1673 | 71.63 |
| 3 | 2129 | 71.85 |
| d) xMSanalyzer | | |
| 3, 0.3 ∪ 3,0.8 | 2677 | 69.66 |

**Additional File 2c.** Feature detection using apLCMS for Sample Set 2 samples from Column B.

| **Parameter Setting** | **Number of aligned *m/z* features** | **median PID between duplicates** |
| --- | --- | --- |
| a) Variation in min.run at min.pres = 0.3 | | |
| 25 | 2938 | 56.43 |
| 20 | 1323 | 49.36 |
| 15 | 1525 | 49.88 |
| 12 (default) | 1713 | 50.90 |
| 9 | 1920 | 52.34 |
| 6 | 2329 | 54.24 |
| 3 | 2938 | 56.43 |
| b) Variation in min.run at min.pres = 0.5 (default) | | |
| 25 | 1059 | 47.24 |
| 20 | 1217 | 46.10 |
| 15 | 1416 | 47.16 |
| 12 (default) | 1573 | 48.73 |
| 9 | 1782 | 50.28 |
| 6 | 2148 | 52.48 |
| 3 | 2768 | 55.15 |
| c) Variation in min.run at min.pres = 0.8 | | |
| 25 | 851 | 44.86 |
| 20 | 1005 | 45.09 |
| 15 | 1186 | 45.39 |
| 12 (default) | 1289 | 46.24 |
| 9 | 1475 | 47.97 |
| 6 | 1856 | 49.48 |
| 3 | 2391 | 52.39 |
| d) xMSanalyzer | | |
| 3, 0.3 ∪ 3,0.8 | 2969 | 50.88 |
