# Supplementary figures and images for: xMSanalyzer: automated pipeline for improved feature detection and downstream analysis of large-scale, non-targeted metabolomics data
Source: BMC Bioinformatics. 2013 Jan 16;14:15. doi: 10.1186/1471-2105-14-15 (PMC3562220; doi:10.1186/1471-2105-14-15)

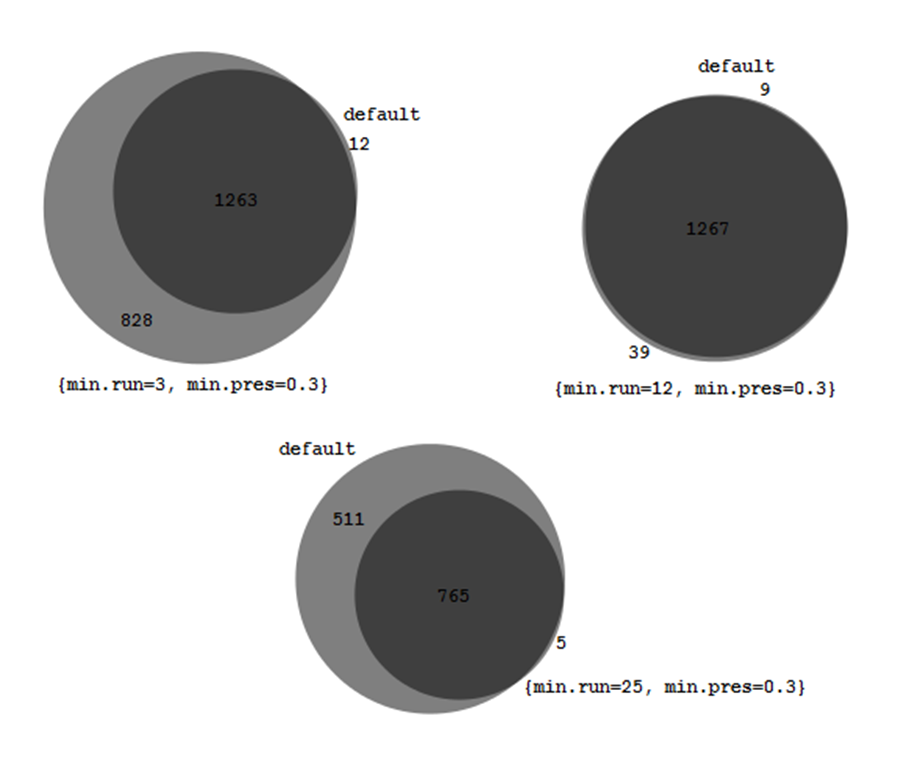

Supplement: Additional file 3 — Venn Diagrams representing overlapping features between the default setting and variations in min.run at min.pres = 0.3. Only unique features atm/ztolerance level of 10 ppm were used to generate Venn diagrams using BioVenn (http://www.cmbi.ru.nl/cdd/biovenn/). [file 1471-2105-14-15-S3.tiff]

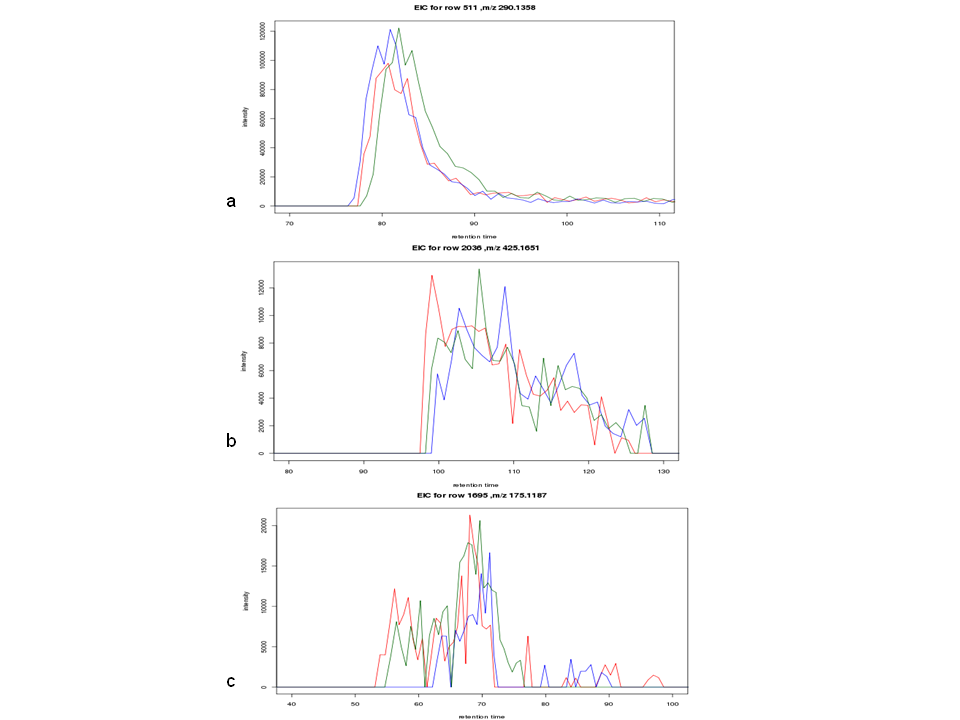

Supplement: Additional file 8 — Extracted ion chromatograms of unique features identified by xMSanalyzer in three biological samples: a) m/z 290.1358 (Gly-Asp-Val); b) m/z 425.16421 (Asp-Tyr-Gln); c) m/z 175.1187 (arginine). [file 1471-2105-14-15-S8.tiff]

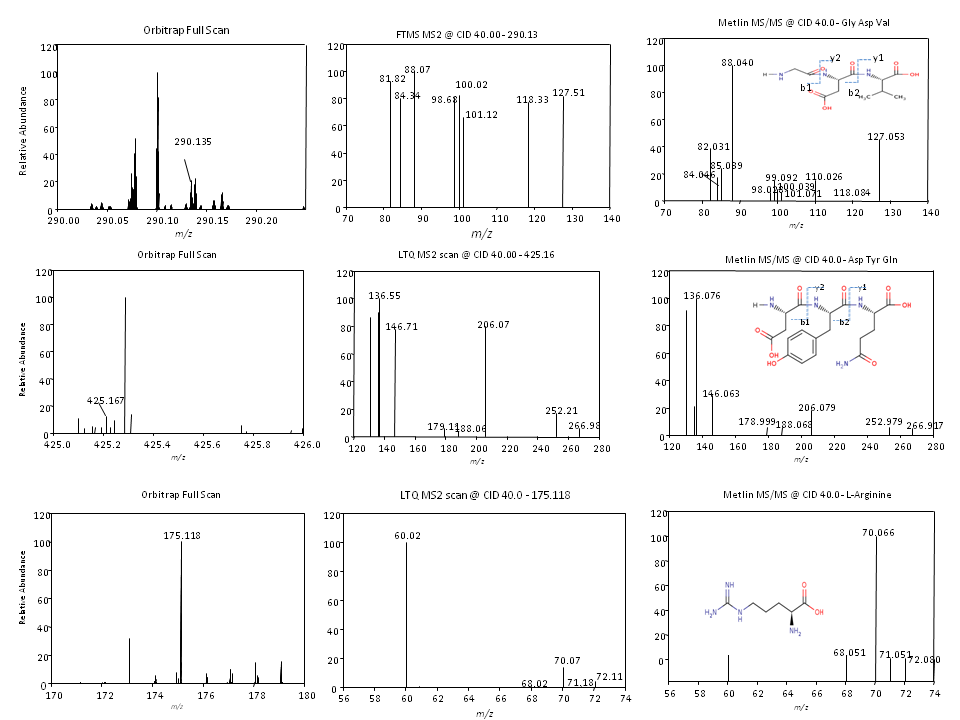

Supplement: Additional file 10 — MS/MS validation results for the metabolites exclusively identified by xMSanalyzer and with matches in Metlin. The first column is the full MS scan, second column is the MS/MS spectrum on LTQ Velos Orbitrap, and the third column shows the corresponding MS/MS spectrum from Metlin’s database. [file 1471-2105-14-15-S10.tiff]
