# Supplementary material for: xMSanalyzer: automated pipeline for improved feature detection and downstream analysis of large-scale, non-targeted metabolomics data
Source: BMC Bioinformatics. 2013 Jan 16;14:15. doi: 10.1186/1471-2105-14-15 (PMC3562220; doi:10.1186/1471-2105-14-15)
Supplement: Additional file 4 — Effect of variation in min.bw and max.bw on feature detection at default settings using a random subset of 10 samples from the Sample Set 1 Column A. [file 1471-2105-14-15-S4.doc]

**Additional File 4.** Effect of variation in min.bw and max.bw on feature detection at default settings using a random subset of 10 samples from the Sample Set 1 Column A.

| **min.bw\max.bw** | **30** | **60** | **NA** |
| --- | --- | --- | --- |
| 1 | 1624 | 1624 | 1624 |
| 5 | 1624 | 1624 | 1624 |
| 30 | 1624 | 1624 | 1624 |
| NA | 1624 | 1624 | 1624 |
