# Supplementary material for: xMSanalyzer: automated pipeline for improved feature detection and downstream analysis of large-scale, non-targeted metabolomics data
Source: BMC Bioinformatics. 2013 Jan 16;14:15. doi: 10.1186/1471-2105-14-15 (PMC3562220; doi:10.1186/1471-2105-14-15)
Supplement: Additional file 5 — Results for merging more than two parameter settings using the Sample Set 1 Column A. [file 1471-2105-14-15-S5.doc]

**Additional File 5.** Results for merging more than two parameter settings using the Sample Set 1 Column A.

| **Parameter** | **Number of features** | **Median Percent Intensity Difference (averaged over all features)** |
| --- | --- | --- |
| 3,0.3 ∪ 3,0.8 ∪ 12,0.5 | 2409 | 35.15 |
| 3,0.3 ∪ 3,0.5 ∪ 3,0.8 | 2434 | 35.01 |
| 3,0.5 ∪ 3,0.8 ∪ 12,0.5 | 2095 | 31.86 |
| 3,0.3 ∪ 3,0.5 ∪ 3,0.8 | 2434 | 35.01 |
| 3,0.3 ∪ 3,0.5 ∪ 3,0.8 ∪ 12,0.5 | 2453 | 34.49 |
