# Supplementary material for: xMSanalyzer: automated pipeline for improved feature detection and downstream analysis of large-scale, non-targeted metabolomics data
Source: BMC Bioinformatics. 2013 Jan 16;14:15. doi: 10.1186/1471-2105-14-15 (PMC3562220; doi:10.1186/1471-2105-14-15)
Supplement: Additional file 6 — Evaluating fitness of parameter combinations based on parameter sensitivity analysis in a) Sample Set 1 Column B; b) Sample Set 2 Column A; and c) Sample Set 2 Column B. [file 1471-2105-14-15-S6.doc]

**Additional File 6a**. Evaluating fitness of parameter combinations based on the parameter optimization scoring function on data from Column B of Sample Set 1.

| **Parameter** | **Number of features** | **Median Percent Intensity Difference (averaged over all features)** | **N-30*median PID**  **(weighs more importance to number of features)** | **N-100*median PID**  **(weighs more importance to quality of features)** |
| --- | --- | --- | --- | --- |
| 12, 0.5 | 1238 | 30.31 | 328.7 | -1793 |
| 3,0.3 | 2112 | 36.85 | 1006.5 | -1573 |
| 3,0.5 | 1800 | 32.55 | 823.5 | -1455 |
| 3,0.8 | 1470 | 27.02 | 659.4 | -1232 |
| 3,0.3 ∪ 3,0.5 | 2146 | 33.75 | 1133.5 | -1229 |
| 3,0.3 ∪ 3,0.8 | 2201 | 33.85 | 1185.5 | -1184 |
| 3,0.5 ∪ 3,0.8 | 1880 | 30.36 | 969.2 | -1156 |
| 3,0.3 ∪ 12,0.5 | 2128 | 34.67 | 1087.9 | -1339 |
| 3,0.5 ∪ 12,0.5 | 1833 | 31.87 | 876.9 | -1354 |
| 3,0.8 ∪ 12,0.5 | 1615 | 28.46 | 761.2 | -1231 |

**Additional File 6b.** Evaluating fitness of parameter combinations based on the parameter optimization scoring function on data from Column A of Sample Set 2.

| **Parameter** | **Number of features** | **Median Percent Intensity Difference (averaged over all features)** | **N-30*median PID**  **(weighs more importance to number of features)** | **N-100*median PID**  **(weighs more importance to quality of features)** |
| --- | --- | --- | --- | --- |
| 12, 0.5 | 1324 | 69.49 | -760.7 | -5625 |
| 3,0.3 | 2618 | 76.14 | 333.8 | -4996 |
| 3,0.5 | 2510 | 74.29 | 281.3 | -4919 |
| 3,0.8 | 2129 | 71.85 | -26.5 | -5056 |
| 3,0.3 ∪ 3,0.5 | 2638 | 71.68 | 487.6 | -4530 |
| 3,0.3 ∪ 3,0.8 | 2677 | 69.66 | 587.2 | -4289 |
| 3,0.5 ∪ 3,0.8 | 2581 | 69.06 | 509.2 | -4325 |
| 3,0.3 ∪ 12,0.5 | 2592 | 72.69 | 411.3 | -4677 |
| 3,0.5 ∪ 12,0.5 | 2503 | 72.23 | 336.1 | -4720 |
| 3,0.8 ∪ 12,0.5 | 2256 | 68.92 | 188.4 | -4636 |

**Additional File 6c.** Evaluating fitness of parameter combinations based on the parameter optimization scoring function on data from Column B of Sample Set 2.

| **Parameter** | **Number of features** | **Median Percent Intensity Difference (averaged over all features)** | **N-30*median PID**  **(weighs more importance to number of features)** | **N-100*median PID**  **(weighs more importance to quality of features)** |
| --- | --- | --- | --- | --- |
| 12, 0.5 | 1573 | 48.73 | 111.1 | -3300 |
| 3,0.3 | 2938 | 56.43 | 1245.1 | -2705 |
| 3,0.5 | 2768 | 55.15 | 1113.5 | -2747 |
| 3,0.8 | 2391 | 52.39 | 819.3 | -2848 |
| 3,0.3 ∪ 3,0.5 | 2927 | 52.28 | 1358.6 | -2301 |
| 3,0.3 ∪ 3,0.8 | 2969 | 50.88 | 1442.6 | -2119 |
| 3,0.5 ∪ 3,0.8 | 2847 | 51.04 | 1315.8 | -2257 |
| 3,0.3 ∪ 12,0.5 | 2911 | 53.17 | 1315.9 | -2406 |
| 3,0.5 ∪ 12,0.5 | 2842 | 52.72 | 1260.4 | -2430 |
| 3,0.8 ∪ 12,0.5 | 2546 | 49.05 | 1074.5 | -2359 |

*Note: Only a subset of results is shown in the table
