# Supplementary material for: xMSanalyzer: automated pipeline for improved feature detection and downstream analysis of large-scale, non-targeted metabolomics data
Source: BMC Bioinformatics. 2013 Jan 16;14:15. doi: 10.1186/1471-2105-14-15 (PMC3562220; doi:10.1186/1471-2105-14-15)
Supplement: Additional file 9 — Summary of MS/MS analysis. [file 1471-2105-14-15-S9.doc]

**Additional File 9**. Summary of MS/MS results

| **m/z** | **Retention Time** | **Average Ion Intensity** | **PID (25th percentile)** | **Metabolite Confirmation** | **DeconMsn**  **Validation** | **Peak Score*** |
| --- | --- | --- | --- | --- | --- | --- |
| 405.2277 | 61 | 323336 | 23.72 | no | No | 1 |
| 219.0968 | 119 | 20540 | 74.15 | no | No | 1 |
| 388.2543 | 220 | 35202 | 8.045 | no | Yes | 1 |
| 219.097 | 491 | 39488 | 23.61 | no | No | 1 |
| 175.1187 | 69 | 32700 | 23.09 | L-arginine | Yes | 1 |
| 334.1386 | 135 | 31901 | 62.88 | no | No | 1 |
| 337.2359 | 222 | 143646 | 14.95 | no | Yes | 3 |
| 340.1607 | 311 | 15771 | 23.37 | no | Yes | 2 |
| 430.2444 | 73 | 11171 | 22.93 | no | Yes | 1 |
| 132.065 | 141 | 13580 | 39.54 | no | No | 1 |
| 409.1734 | 294 | 27547 | 55.69 | no | Yes | 1 |
| 366.1335 | 80 | 23048 | 25.06 | no | Yes | 1 |
| 438.2346 | 89 | 57127 | 9.553 | no | Yes | 1 |
| 300.1288 | 98 | 133275 | 38.88 | no | No | 1 |
| 389.2494 | 100 | 55518 | 25.09 | no | Yes | 2 |
| 290.1358 | 104 | 24110 | 11.92 | Gly Asp Val | Yes | 1 |
| 425.1651 | 113 | 103482 | 25.79 | Asp Tyr Gln | Yes | 1 |

*Peak Score: 0=no peak; 1=single peak; 2=multiple peaks (>= 2 RT); 3=solvent peak (broad)
