# Supplementary material for: xMSanalyzer: automated pipeline for improved feature detection and downstream analysis of large-scale, non-targeted metabolomics data
Source: BMC Bioinformatics. 2013 Jan 16;14:15. doi: 10.1186/1471-2105-14-15 (PMC3562220; doi:10.1186/1471-2105-14-15)

Additional File 11. MS/MS validation results for the 8 putative metabolites identified using DeconMSn. The first row shows the elution profile of the feature, middle row shows the full MS scan, and the last row shows the deconvoluted MS/MS spectrum obtained from DeconMSn.

1) m/z 388.254

Elution profile

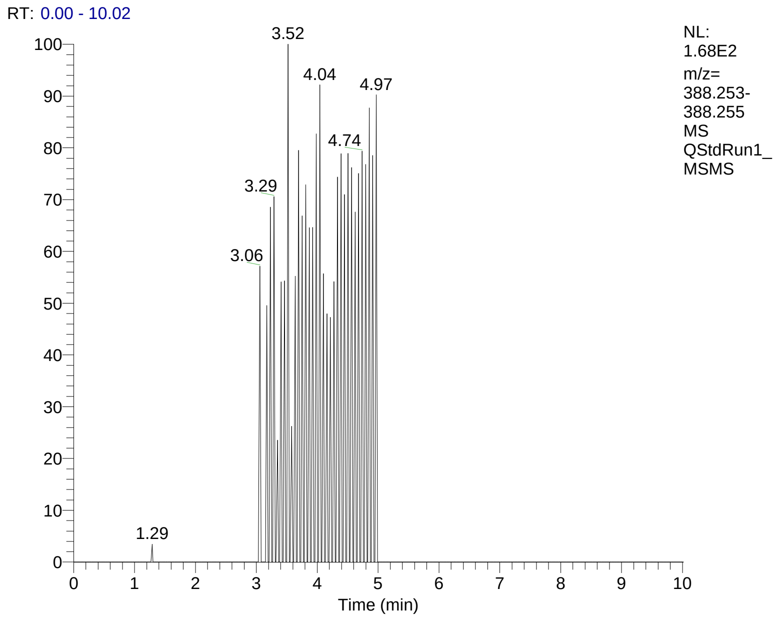

MS, FTMS

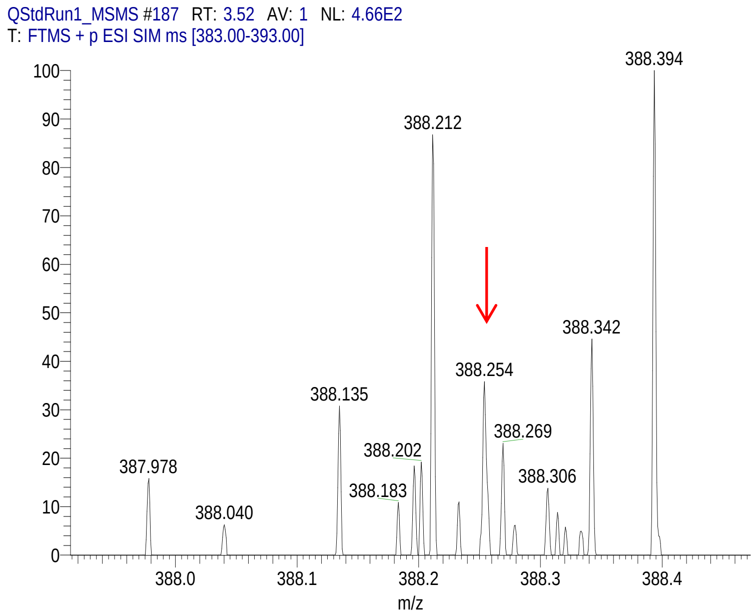

Deconvoluted MS/MS
CID in Ion trap

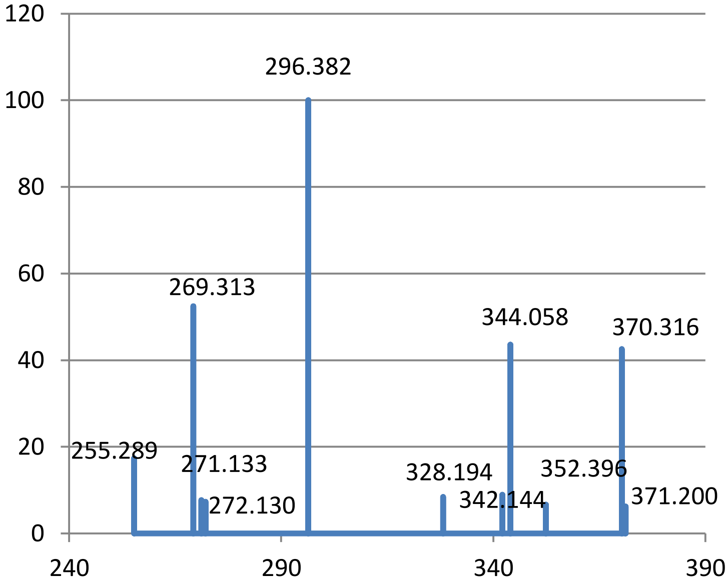

2) m/z 430.244

Elution profile

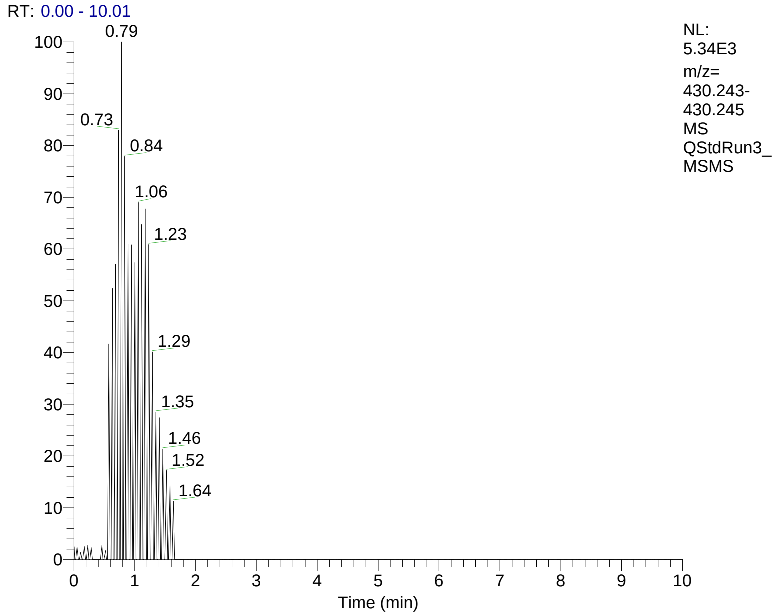

MS, FTMS

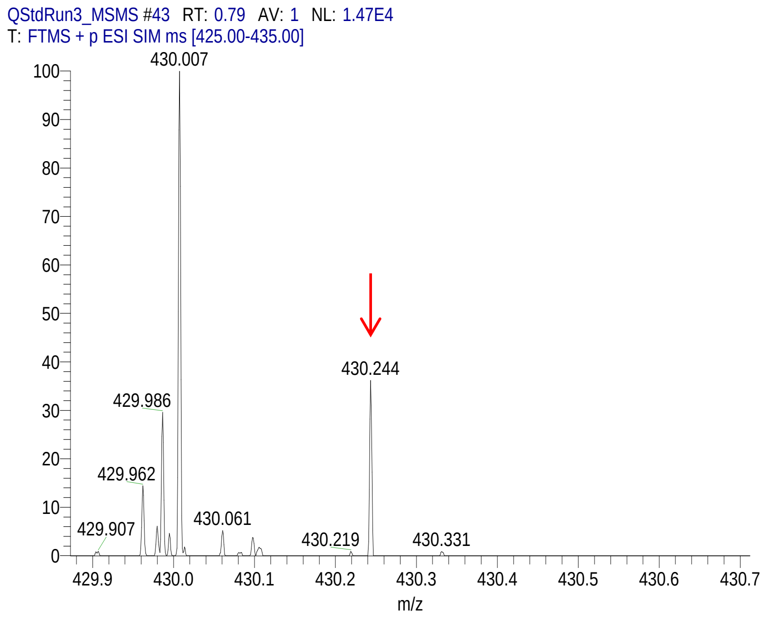

Deconvoluted MS/MS
CID in Ion trap

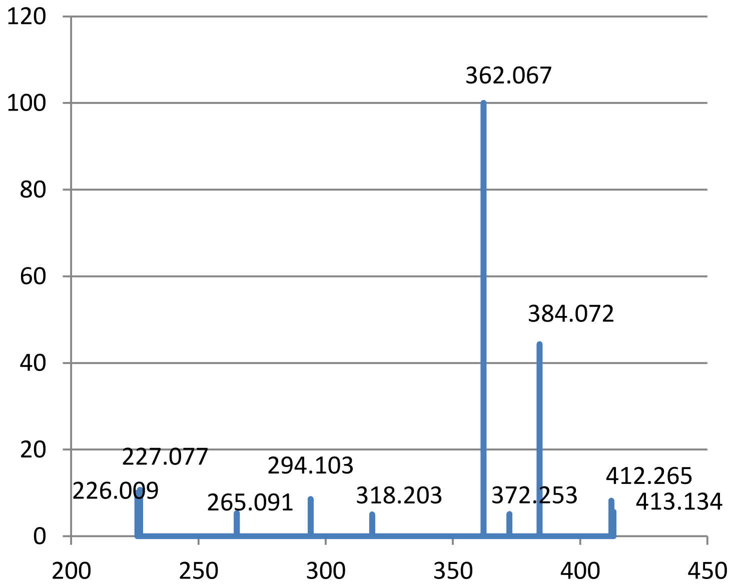

3) m/z 337.235

Elution profile

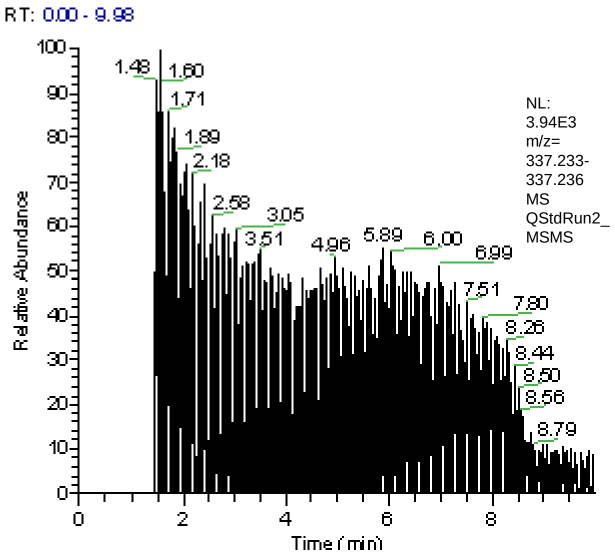

MS, FTMS

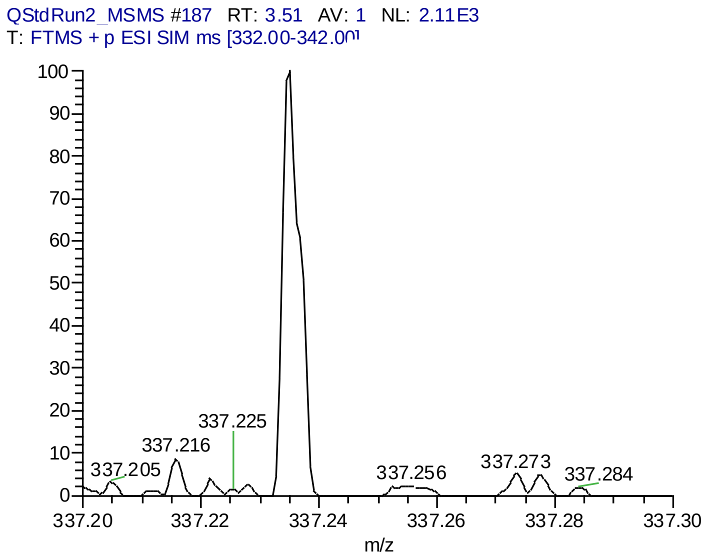

Deconvoluted MS/MS
CID in Ion trap

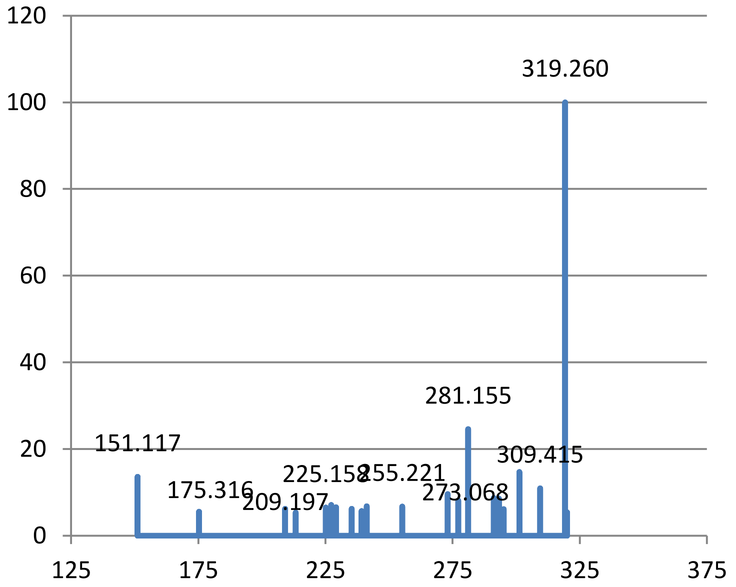

4) m/z 340.160

Elution profile

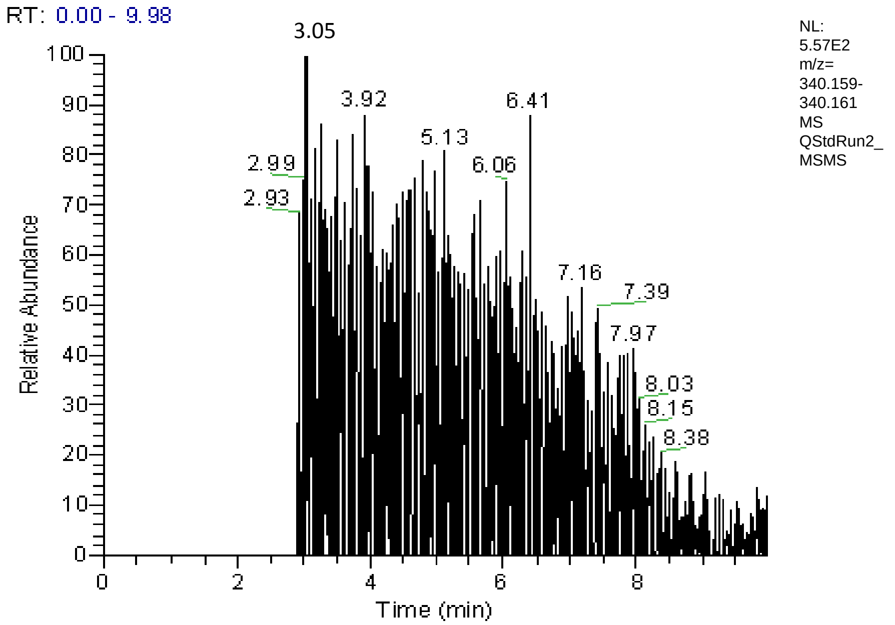

MS, FTMS

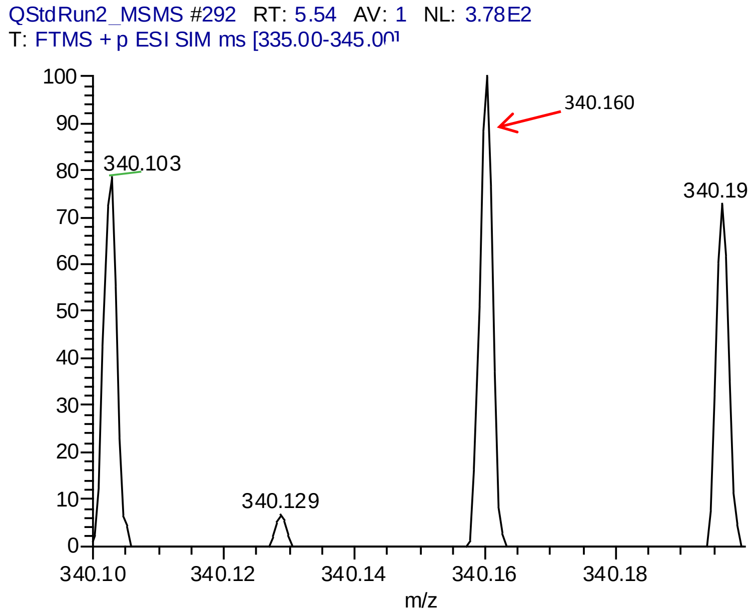

Deconvoluted MS/MS
CID in Ion trap

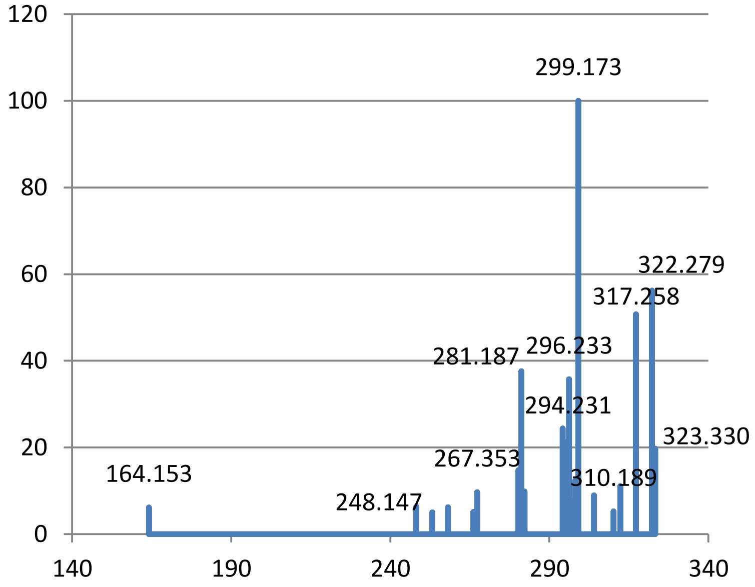

5) m/z 409.173

Elution profile

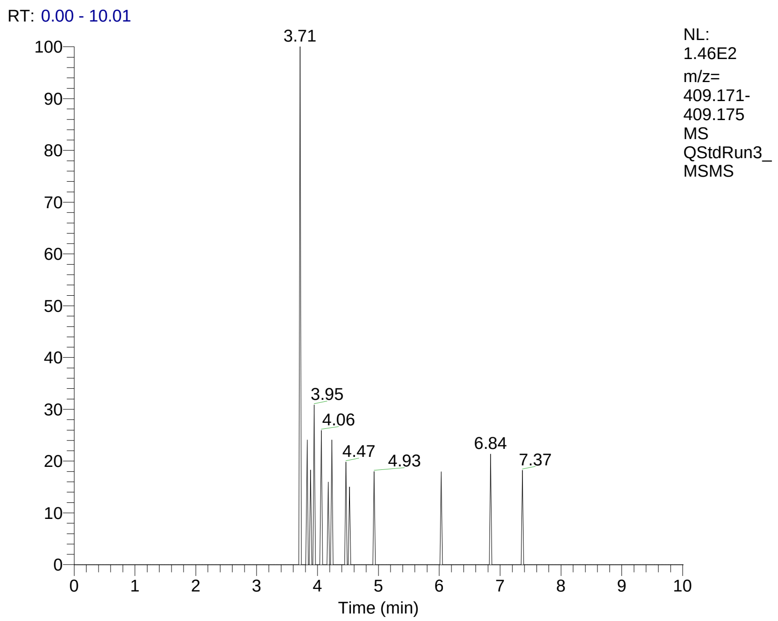

MS, FTMS

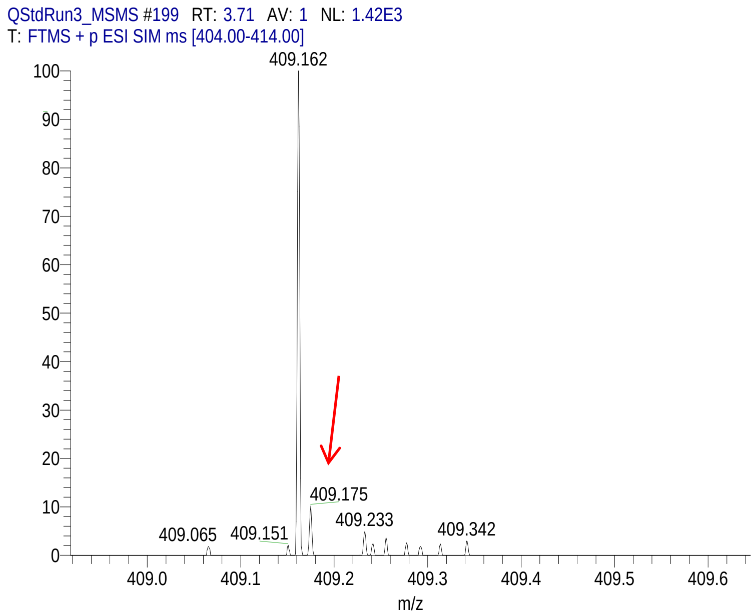

Deconvoluted MS/MS
CID in Ion trap

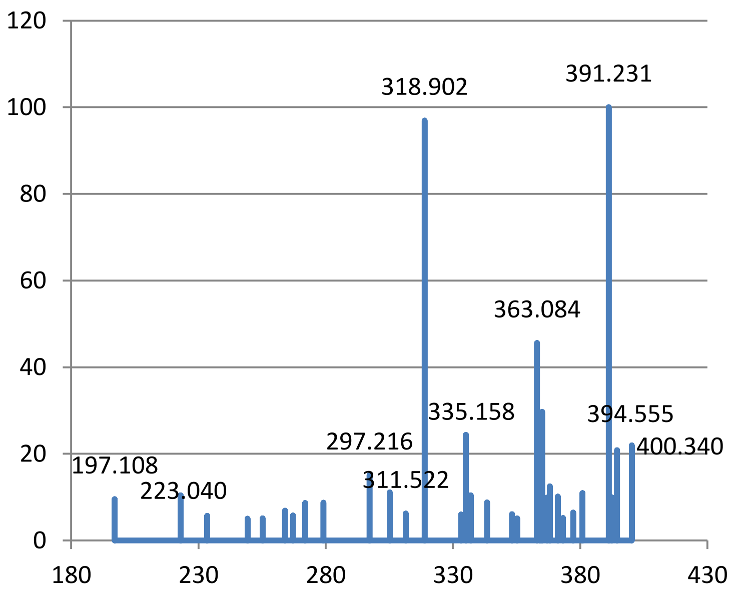

6) m/z 366.133

Elution profile

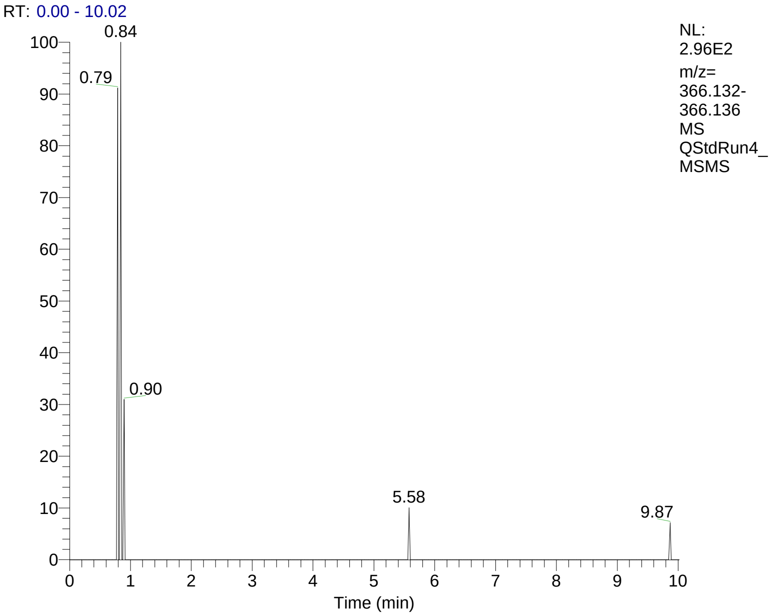

MS, FTMS

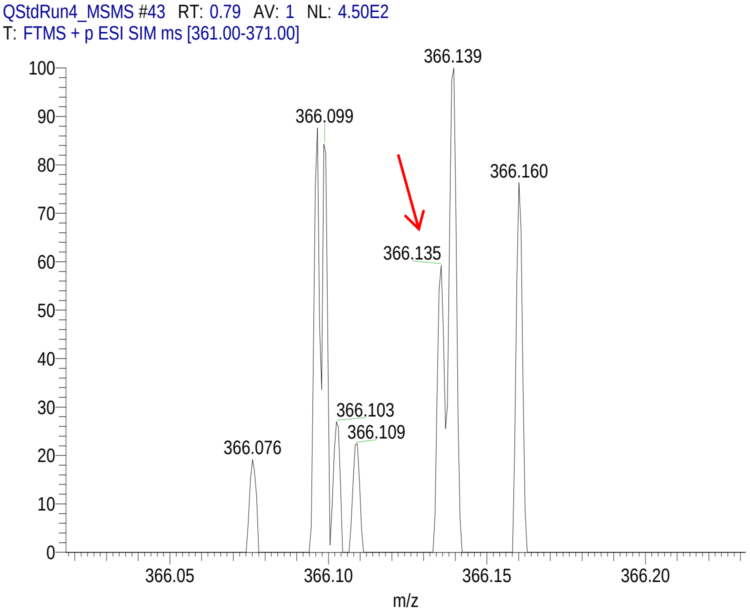

Deconvoluted MS/MS
CID in Ion trap

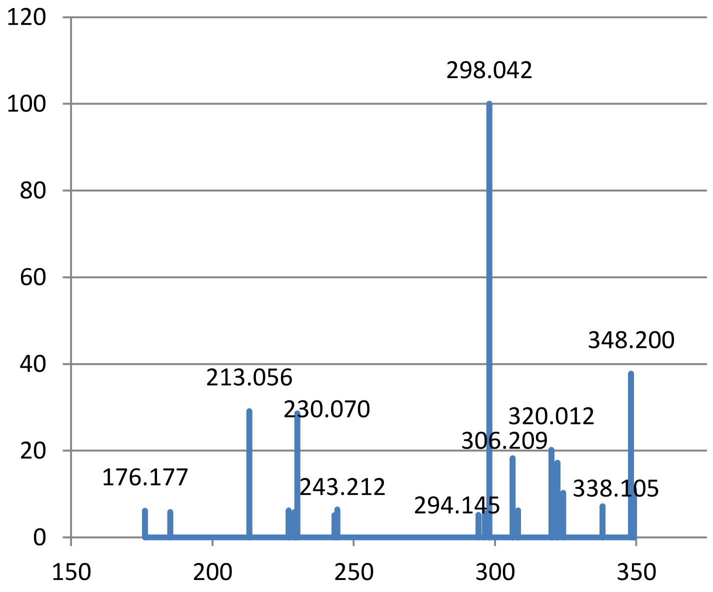

7) m/z 438.234

Elution profile

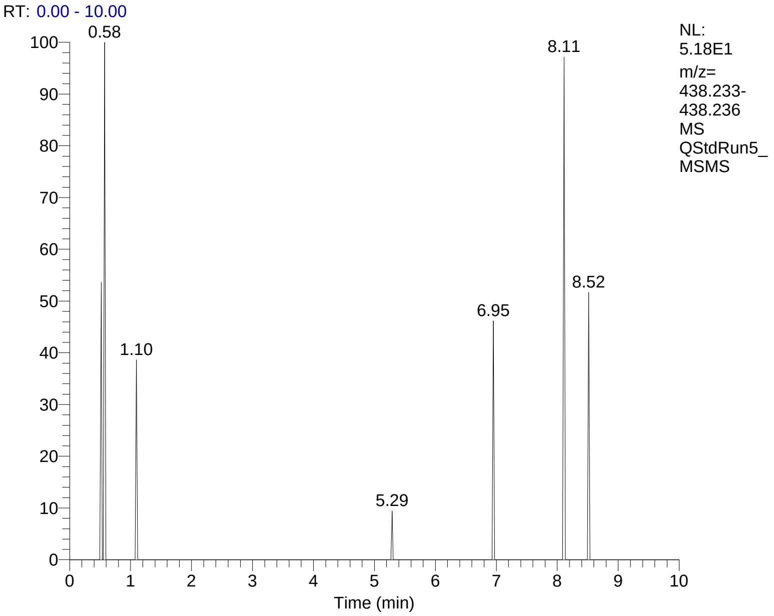

MS, FTMS

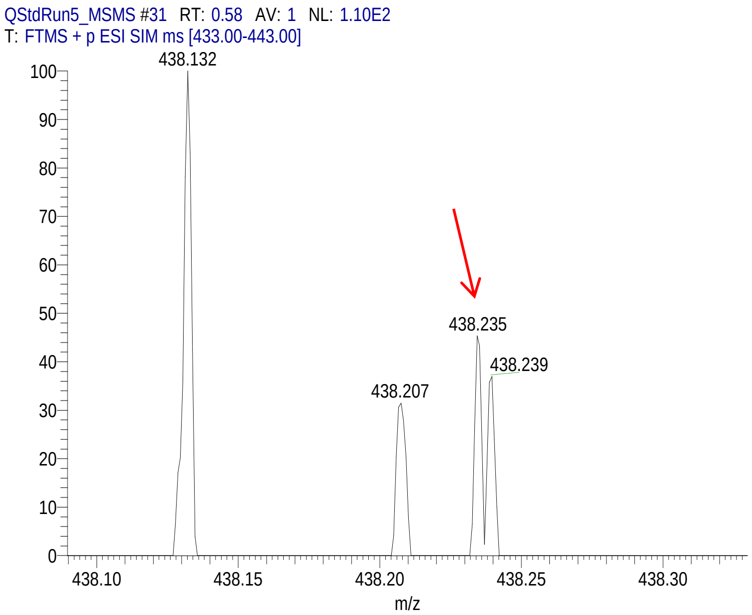

Deconvoluted MS/MS
CID in Ion trap

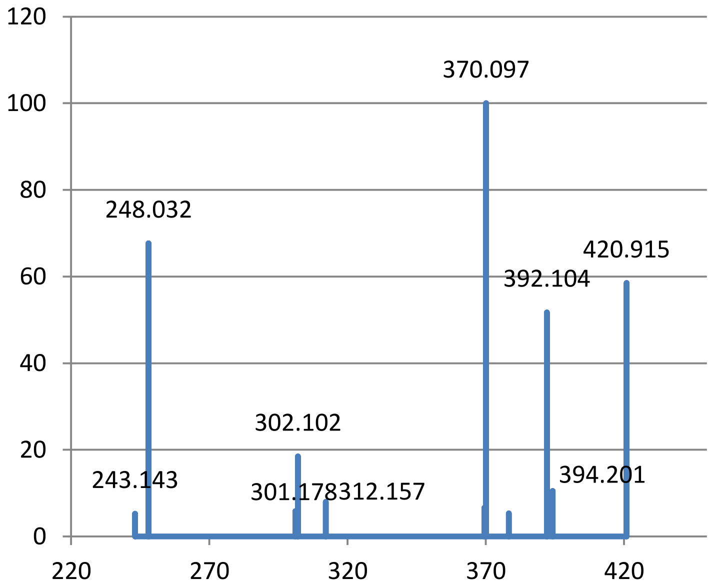

8) m/z 389.249

Elution profile

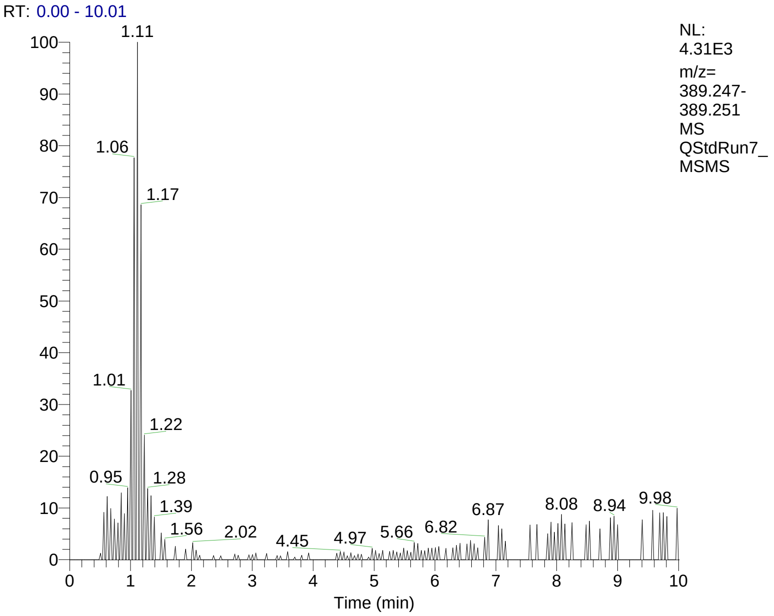

MS, FTMS

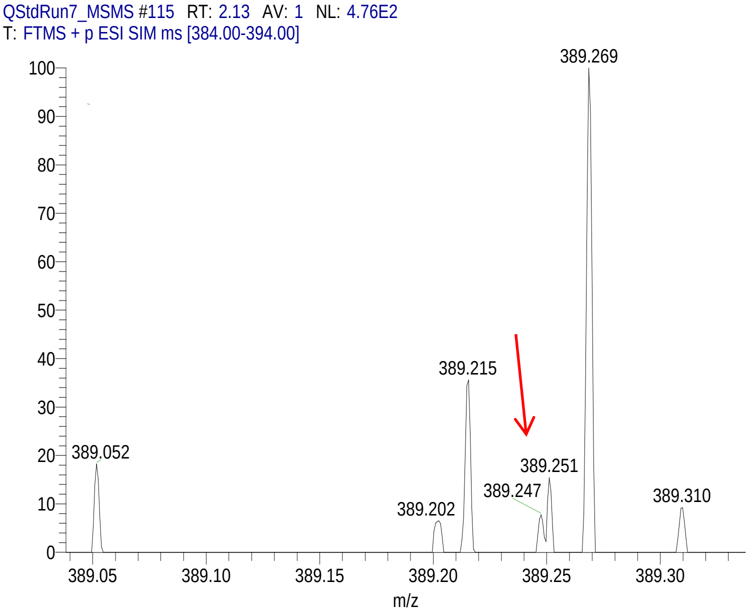

Deconvoluted MS/MS
CID in Ion trap

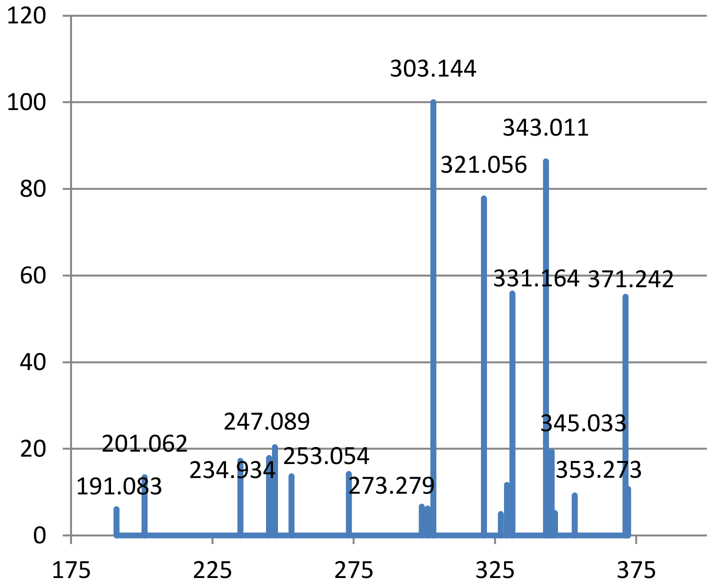

Supplement: Additional file 11 — MS/MS validation results for the 8 putative metabolites identified using DeconMSn. The first row shows the elution profile of the feature, middle row shows the full MS scan, and the last row shows the deconvoluted MS/MS spectrum obtained from DeconMSn. [file 1471-2105-14-15-S11.pdf]
